# Effect of IV ferric carboxy maltose for moderate/severe anemia: a systematic review and meta-analysis

**DOI:** 10.3389/fmed.2024.1340158

**Published:** 2024-02-09

**Authors:** Mahalaqua Nazli Khatib, Anju Pradhan Sinha, Shilpa Gaidhane, Shilpa Upadhyay, Nikita Waghmare, Abhishek Anil, Deepak Saxena, Shailendra Sawleshwarkar, Padam Prasad Simkhada, Abhay Gaidhane, Zahiruddin Syed Quazi

**Affiliations:** ^1^Global Evidence Synthesis Initiative (GESI), Division of Evidence Synthesis, School of Epidemiology and Public Health, Jawaharlal Nehru Medical College, Datta Meghe Institute of Higher Education and Research, Wardha, Maharashtra, India; ^2^Division of Reproductive, Maternal and Child Health, Indian Council of Medical Research Headquarters, New Delhi, India; ^3^Centre of One Health Research, Department of Medicine, Jawaharlal Nehru Medical College, Datta Meghe Institute of Higher Education and Research, Wardha, Maharashtra, India; ^4^Global Consortium of Public Health Research, Jawaharlal Nehru Medical College, Datta Meghe Institute of Higher Education and Research, Wardha, Maharashtra, India; ^5^i-Health Consortium, Division of Evidence Synthesis, School of Epidemiology and Public Health, Jawaharlal Nehru Medical College, Datta Meghe Institute of Higher Education and Research, Wardha, Maharashtra, India; ^6^Department of Pharmacology, All India Institute of Medical Sciences (AIIMS), Jodhpur, Rajasthan, India; ^7^Department of Epidemiology, Indian Institute of Public Health, Gandhinagar, Gujarat, India; ^8^Faculty of Medicine and Health, Sydney Medical School, The University of Sydney Institute for Infectious Disease (Sydney ID), University of Syndey, Camperdown, NSW, Australia; ^9^School of Human and Health Sciences, Global Health at the University of Huddersfield, Huddersfield, United Kingdom; ^10^Stepping Stones, Jawaharlal Nehru Medical College, Datta Meghe Institute of Higher Education and Research, Wardha, Maharashtra, India; ^11^South Asia Infant Feeding Research Network, Global Health Academy, Jawaharlal Nehru Medical College, Datta Meghe Institute of Higher Education and Research, Wardha, Maharashtra, India

**Keywords:** ferric carboxymaltose, iron deficiency anemia, intravenous iron supplementation, moderate to severe anemia, hemoglobin

## Abstract

**Introduction:**

Anemia remains a prevalent global health issue with varying severity. Intravenous iron supplementation, particularly with ferric carboxymaltose (FCM), has appeared as a possible therapeutic intervention for individuals with moderate to severe anemia. The study aimed to assess the efficacy and safety of ferric carboxymaltose (FCM) in reducing anemia.

**Methods:**

We searched electronic databases, registries, websites, e-libraries, reference lists of reviews, citations, etc. We included randomized control trials (RCTs), non-RCTs, and single-arm studies, while observational studies, case series, and case studies were excluded. Two reviewers independently screened the studies and extracted the data. We included studies of moderate-to-severely anemic Indians and excluded Indians with other comorbidities. We assessed the risk of bias and the overall quality of evidence (QoE) using GRADE GDT.

**Result:**

We identified 255 studies and included 14 studies (11 RCT, one non-RCT, and two single-arm studies) with 1,972 participants for qualitative analysis and 10 studies in the meta-analysis. All the included studies detailed the use of FCM for anemia. The primary outcomes assessed in the included studies were anemia, hemoglobin, and adverse events. The outcomes assessed ranged from 2 weeks to 12 weeks. The risk of bias varied across different studies with different outcomes. FCM is consistent with a fewer number of adverse events as compared to other interventions and provides “moderate” to “very low” QoE.

**Conclusion:**

A slow single infusion of 1 gram of FCM is well-tolerated, safe, and effective in treating iron deficiency anemia (IDA) and surpasses other interventions (Iron Sucrose Complex (ISC), Iron sucrose, and ferrous ascorbate) in elevating hemoglobin levels and replenishing iron stores.

**Systematic Review Registration:**

https://www.crd.york.ac.uk/prospero/display_record.php?RecordID=459363, CRD42023459363.

## 1 Introduction

Anemia is indicated by a deficiency in the number of red blood cells or below-average hemoglobin levels within these cells (1). This condition presents a noteworthy public health challenge, influencing not just individual well-being but also significantly impacting societal and economic advancement (2). As per WHO 2023 estimates, 42% of children under five and 40% of pregnant women are anemic globally (1). According to National Family Health Survey (NFHS-5) data organized in India (between 2019 and 2021), 57% of women and 25% of men in the age group of 15–49 years are anemic in India (3). Despite the availability of treatments and guidelines (Anemia Mukt Bharat), the slightest improvement is observed in the anemia status in India (4).

Causes of anemia can often be distinct but frequently coexist. The primary cause of anemia encompasses nutritional deficiencies, hemoglobinopathies, and infectious diseases (like malaria, tuberculosis, HIV, and parasitic infections). While it’s mostly presumed that around 50% of anemia cases stem from iron deficiency, this ratio might vary among different population groups and regions (5). Acknowledging the multifaceted nature of this ailment, rectifying anemia necessitates a comprehensive strategy (5). An integrated approach is crucial to combat it effectively, identifying and mitigating contributing factors.

Iron deficiency typically evolves gradually, often without evident symptoms or clinical manifestations. As iron reserves are gradually exhausted, iron availability to tissues diminishes, resulting in symptomatic anemia (6). This includes fatigue, weakness, dizziness and shortness of breath (6).

Although anemia can occur at any stage of life, pregnant women and young children are more inclined. The health effects of anemia include a high risk of maternal and child mortality, a negative impact on children’s cognitive development, physical development, physical performance, and increased susceptibility to infections in adults. Anemia during the antepartum period distinctly impacts both maternal and fetal well-being. It is intricately associated with more significant morbidity and risk of several challenges throughout pregnancy, such as greater susceptibility towards infection, increased need for blood transfusion during delivery, cardiovascular complications, intrauterine growth retardation, preterm delivery, and perinatal mortality and morbidity (7, 8). During the first trimester, IDA harms fetal growth more than during late pregnancy (6, 7, 9). Anemia during the post-partum period inflicts a significant disease burden at a vital phase of maternal–infant interaction and may result in developmental impairments in afflicted mothers’ newborns (6).

In regions where Iron Deficiency Anemia (IDA) is the predominant cause of anemia (especially in low-income contexts), supplementary iron is often administered through supplements to vulnerable groups. Strategies like fortifying food and diversifying diets to enhance iron consumption emerge as crucial and sustainable methods to combat IDA within the broader aspects. However, a comprehensive approach incorporating iron interventions alongside other strategies becomes imperative when anemia is not solely attributed to iron deficiency.

The primary approach to addressing it involves oral or intravenous (IV) iron supplementation, targeting the underlying cause of IDA, and restoring iron levels to normal. The initial method of choice is oral iron supplementation. However, challenges related to compliance and the potential for iron depletion undermine the efficacy of oral iron treatment (10). Oral interventions prove inadequate in cases of moderate to severe anemia, necessitating prompt elevation of hemoglobin levels and rapid iron store replenishment. Instead, expedited remedies like parenteral therapies become imperative (11). Notably, parenteral options, including intravenous iron preparations, facilitate swifter iron restoration compared to oral methods, and their tolerability during pregnancy is notable. Among the commonly utilized parenteral preparations, Iron Sucrose Complex (ISC) and iron dextran are dosed according to the level of iron deficiency. Nevertheless, it’s crucial to acknowledge that intravenous iron dextran formulations risk allergic reactions, whereas intravenous iron polymaltose mandates a lengthier infusion time.

Ferric carboxymaltose (FCM) is a third-generation intravenous dextran-free, intravenous iron formulation given in a single dose over a small duration, which overcomes the limitations of existing treatments and has a greater capacity for restoring iron (12). It minimizes the dose frequency but also has few drug-related side effects. Clinical findings have shown intravenous FCM’s efficacy is effective in spanning conditions like uterine bleeding, post-partum iron deficiency anemia, inflammatory bowel disease, and chronic kidney disease, irrespective of hemodialysis (13).

Ferric carboxymaltose is an innovative iron complex composed of a ferric hydroxide core, facilitating controlled iron delivery to reticuloendothelial cells and subsequently to iron-binding proteins like ferritin and transferring (13). This complex minimizes the risk of releasing excessive ionic iron into the serum. It is swiftly eliminated through the bloodstream and predominantly circulated to the bone marrow, liver, and spleen (13). This deliberate gradual release mechanism contributes to the low toxicity of FCM, establishing a substantial safety margin between standard and lethal doses. Furthermore, the FCM formulation’s neutral pH and physiological osmolarity permit the administration of elevated doses with favorable local tolerance. As long as the iron dosage is tailored to the patient’s needs, the likelihood of FCM-induced toxicity during clinical use remains relatively low. Additionally, FCM stands out for its absence of dextran ferumoxytol and iron isomaltose, minimizing the risk of dextran-induced anaphylactic reactions. Its exceptional safety profile, remarkably low immunogenicity, and often singular-dose regimen enhance its cost-effectiveness, particularly in most cases.

Though studies (8, 14–21) have demonstrated positive and encouraging effects of FCM in anemic individuals, there exists an urge to generate evidence for patients, practitioners and policymakers to determine the potential integration of intravenous FCM in the management of moderate to severely anemic individuals and, if data permits, to figure out the most appropriate drug dosage for this group of patients. Therefore, we plan to systematically review existing literature that reports intravenous FCM’s effectiveness in treating moderate-to-severe anemia. This systematic review aimed to assess the efficacy and safety of ferric carboxymaltose in reducing anemia.

## 2 Methods

The systematic review was conducted using a standard methodology suggested in the Cochrane Handbook of Systematic Reviews (22). The protocol of this systematic review was registered in Prospero. The registration number of the proposed protocol is https://www.crd.york.ac.uk/prospero/display_record.php?ID=CRD42023459363. This systematic review was funded by Indian Council of Medical Research (ICMR), India.

### 2.1 Inclusion and exclusion criteria

#### 2.1.1 Types of studies

Inclusion criteria encompassed Randomized Control Trials (RCTs), non-RCTs, and single-arm studies while observational studies, case series, and case studies were excluded. Full journal publication was mandatory for inclusion, though extended abstracts of otherwise unpublished clinical trials were accepted.

#### 2.1.2 Types of participants

Studies based on moderate to severely anaemic Indians irrespective of age groups, gender, ethnicity, educational status, community or setting, other socio-demographic factors, type of anaemia (nutritional deficiencies, such as iron, folate, vitamins B_12_ and A; haemoglobinopathies; infectious diseases, such as malaria, tuberculosis, HIV and parasitic infections) were incorporated in this systematic review. Studies done in South-East Asia or at the global level were considered if they provided data separately for the Indian inhabitants. Studies confined to Indians with other comorbidities were excluded from the review.

#### 2.1.3 Types of interventions

Studies in which intravenous ferric carboxymaltose injection was administered to moderately or severely anaemic Indians irrespective of dose, frequency and duration were incorporated in this systematic review.

#### 2.1.4 Types of comparisons

The following comparisons were made in the review:

1. FCM versus placebo
2. FCM versus no treatment
3. FCM versus alternative experimental treatment modality (Iron sucrose or other);
4. FCM in combination with other treatments versus FCM treatment alone.

#### 2.1.5 Types of outcomes

The following outcomes were considered in the review:

Primary outcomes:

1. Anaemia
2. Haemoglobin
3. Adverse events: Adverse reaction was considered if the patient experienced any reaction during infusion or after drug administration. It was assessed as a dichotomous outcome with a number of participants who reported adverse events.

Secondary outcomes:

1. Iron profile (such as serum iron, serum ferritin, transferrin saturation, Total Iron Binding Capacity).

Reporting of these outcome measures did not form part of the criteria for including studies in a review.

### 2.2 Search methods for identification of studies

The Cochrane Central Register of Controlled Trials (CENTRAL) (via the Cochrane Library), MEDLINE (via PubMed) Medical subject headings (MeSH) or equivalent and text-word terms were used in order to search bibliographic databases without language restrictions. We preferred studies published in English. Searches were tailored to individual databases. Furthermore, we searched the metaRegister of controlled trials (mRCT),^1^ clinicaltrials.gov,^2^ and the WHO International Clinical Trials Registry Platform (ICTRP)^3^ for ongoing trials. Moreover, we examined the reference lists of retrieved articles and conducted hand searches of abstracts from relevant conferences. To uncover additional literature pertinent to the review, we engaged field experts for insights into unpublished and ongoing trials.

#### 2.2.1 CENTRAL search strategy

Search Name:ICMR_Ferric carboxymaltose for anemia

Last Saved:09/01/2023 13:29:47

Comment:

IDSearch

#1“ferric carboxymaltose”

#2“ferric carboxy-maltose”

#3“ferric carboxy-maltose”

#4“iron carboxymaltose”

#5“iron carboxy maltose”

#6“iron carboxy-maltose”

#7“ferric compounds”

#8“iron compounds”

#9“iron complex*”

#10“Iron polymaltose”

#11“polynuclear iron”

#12Ferinject*

#13Injectafer*

#14VIT-45

#15“VIT 45”

#16MeSH descriptor: [Ferric Compounds] explode all trees

#17#1 OR #2 OR #2 OR #3 OR #4 OR #5 OR #6 OR #7 OR #8 OR #8 OR #9 OR #10 OR #11 OR #11 OR #12 OR #13 OR #14 OR #15 OR #16

#18anemi*

#19anaemi*

#20hemoglobin

#21hemoglobin

#22MeSH descriptor: [Anemia] explode all trees

#23MeSH descriptor: [Hemoglobins] explode all trees

#24#18 OR #19 OR #20 OR #21 OR #22 OR #23

#25India*

#26MeSH descriptor: [India] explode all trees

#27#23 OR #26

#28#17 AND #24 AND #27

### 2.3 Selection of studies

Two reviewers (SU and AA) independently screened the articles retrieved from the searches using the Rayyan online screening tool and determined eligibility by reading the abstract of each study. Subsequently, the review authors eliminated studies that failed to satisfy inclusion criteria and acquired full copies of the remaining studies. Two reviewers (SU and AA) read these studies independently to examine relevant studies, and a third author (MNK) was adjudicated in the event of disagreement. The studies were not anonymised before the assessment. A Preferred Reporting Items for Systematic Reviews and Meta-Analyzes (PRISMA) flow chart was incorporated in the review for a comprehensive overview (23). Notably, the studies included in this review were, irrespective of measured outcome data, reported in a “usable” way.

### 2.4 Data extraction and management

Three review authors (AA, SU and NW) independently extracted data utilizing standardized form, ensuring consistency. Details of the study, participants, intervention and outcomes were extracted and populated in the ‘Characteristics of Studies Table’. The multiple reports of the same study were amalgamated, thus treating each study as the primary unit of focus rather than individual reports.

### 2.5 Assessment of risk of bias in included studies

In each study, two authors (AG and DS) independently assessed the risk of bias, referencing the criteria outlined in the Cochrane Handbook for Systematic Reviews of Interventions, with any disparities fixed by discussion. We completed a “Risk of Bias” table for each, including using the “Risk of bias 2” (RoB 2) (24) tool for RCTs and ROBINS-I tool (25) for non-RCTs.

### 2.6 Measures of treatment effect

We employed fixed-effect and random-effects models that gage the comprehensive effect’s direction, size, and consistency. Risk ratios (RR) along with 95% confidence intervals (CI) were calculated for dichotomous variables, while mean differences (MD) with 95% CI were used for continuous data when measurements were consistent across studies. Standardized mean differences (SMD) were applied for conceptually similar results with varied measurement scales. Comprehensive records of means and standard deviations were noted. Imputation was performed in cases lacking sufficient information for calculating standard deviations of changes.

When published data were missing, incomplete or inconsistent with RCT protocols, we pursued additional information from the original authors/manufacturers. We emailed authors to solicit the necessary details for studies presenting data discrepancies.

The clinical heterogeneity was assessed using the Chi2 test (*p* value <0.1 for statistical significance) and quantified with the I^2^ statistic. Heterogeneity was considered considerable if I2 exceeded 75%, substantial between 50 and 90%, moderate between 30 and 60%, and mild if below 40%. In cases of statistical heterogeneity (I^2^ ≥ 50%), we conducted prespecified subgroup analysis and employed a random-effects model to explore potential causes. Subgroup analyzes were performed based on ferric carboxymaltose administration duration.

In the case of 10 or more included studies, we intended to perform the funnel plot test to detect reporting bias. We also investigated potential and feasible sources of asymmetry in the funnel plot (Supplementary material).

We used the statistical package RevMan 5.4 for analysis, conducting a meta-analysis only when participants, interventions, comparisons, and outcomes were identical, ensuring a clinically meaningful and relevant answer.

As recommended by ‘The Cochrane Handbook’, chapter 4.6.6., and the ‘GRADE Handbook for grading the quality of evidence and strength of recommendations’ (26), we included a ‘Summary of findings’ (SoF) table. SoF tables were presented for comparisons of Hemoglobin, serum ferritin, and adverse events between FCM and alternative experimental treatments (ISC, iron sucrose, ferrous ascorbate). Utilizing the GRADE gdt system, two review authors assessed the overall evidence quality for each outcome and presented findings in the SoF tables. Decisions to downgrade study quality were substantiated through footnotes.

### 2.7 The grades of evidence as per the GRADE working group are

#### High quality

We are very confident that the true effect lies close to that of the estimate of the effect.

#### Moderate quality

We are moderately confident in the effect estimate: The true effect is likely to be close to the estimate of the effect, but there is a possibility that it is substantially different.

#### Low quality

Our confidence in the effect estimate is limited: The true effect may be substantially different from the estimate of the effect.

#### Very low quality

We have very little confidence in the effect estimate: the true effect is likely to be substantially different from the estimate of effect (26).

## 3 Results

**Figure 1:**
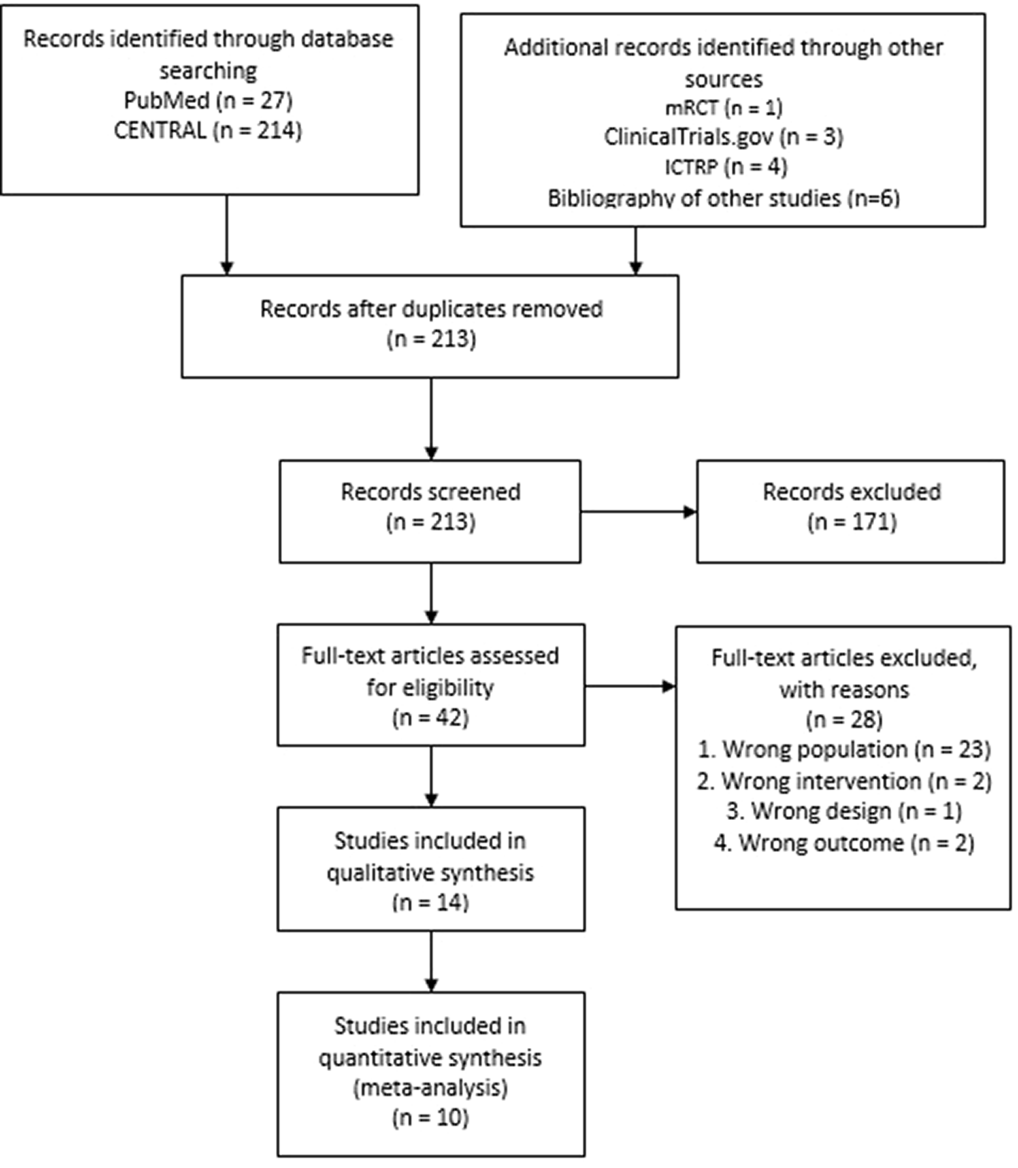
PRISMA flow diagram.

A total 255 records were retrieved from electronic sources. After removing the duplicates, the search yielded 213 records. We discarded 171 records in initial screening based on title and abstracts. We assessed full text of the remaining 42 articles for eligibility and excluded 28 articles based on wrong population (*n* = 23), wrong intervention (*n* = 2), and wrong design and outcome (*n* = 3). Finally, we included the remaining 14 studies in qualitative synthesis and ten in the meta-analysis. We presented the selection process as a PRISMA Flow diagram in Figure 1.

We found 14 studies (11 RCT, one non-RCT and two single-arm studies) which fulfilled our inclusion criteria (1,972 participants). All the studies were single-centric and carried out in different states of India such as New Delhi (15, 27), Cuttack (28), Kolkata (18), Jammu & Kashmir (19), Maharashtra (8), Central India (29), Gujarat (20), Rajasthan (14), North-eastern region (30), Haryana (16, 17), Karnataka (31), and one study (21) conducted in India, however, did not mention the place of research. The care settings reported were hospitals (Tertiary-care hospitals and sub-district hospitals), teaching Institutes and research centers.

All the studies included adults, with the mean age of the study participants ranging from 18 to 40 years. All the studies recruited people with moderate to severe anemia. Eight studies were conducted on pregnant women (8, 14–16, 18–21), five on post-partum women (17, 19, 28–30), two on females with menorrhagia (19, 27), and two studies on women of reproductive age groups (19, 31). None of the studies were conducted on men. All studies except two that failed to specify the type of anemia (16, 17) included participants with IDA.

All the included studies detailed the use of FCM for anemia. Eleven studies (8, 14, 15, 18–21, 27–30) compared FCM versus ISC; among them, one study compared FCM versus ISC as well as intramuscular (IM) Iron sorbitol (21), and one study compared FCM versus ISC as well as oral ferrous ascorbate (28). One study (31) only compared FCM versus ferrous ascorbate. Two studies (16, 17) were single-armed studies that assessed only the effect of FCM. All included studies except one study by Dakhale et al., 2021 (29) administered maximum single dose of 1 g diluted in either 100 mL (16, 17, 21, 29) or 200 mL (8, 15, 20, 27) or 250 mL (19, 28, 30, 31) of 0.9% normal saline solution as drip infusion for 15 min (16, 17, 21, 27, 28, 30, 31) or 30 min (8, 15, 20) or 45 min (19) at baseline. Dakhale et al. (29) administered 500 mg FCM diluted in 100 mL normal saline. In five studies (14, 15, 19, 27, 28), subsequent doses of FCM, if needed were administered not more than one infusion every week (14, 15, 19, 27, 28). Single doses were administered in three included studies (17, 21, 31).

In the comparison group, ISC was slowly infused intravenously as 200 mg (8, 14, 19–21, 29, 30) or 300 mg (15, 27, 28) in 100 mL (8, 20, 21, 29) or 200 mL (15, 19, 27, 30) or 300 mL (28) of 0.9% NS over 15–30 min (8, 15, 19–21, 28, 30) or two hours (27) or daily (21) or on alternate days (8, 14, 19, 28, 30) or twice weekly (15, 20, 27), or after 2 weeks (29).

Hemoglobin was assessed in all the included studies. Except for the two included studies (27, 31), all other studies assessed the serum ferritin levels. Most of the studies except four studies (15, 17, 19, 27) reported adverse events. The outcomes assessed ranged between 2 weeks to 12 weeks. We have presented characteristics of included studies in Table 1.

**Table 1:** Characteristics of all included studies.

SN	Study ID	Study design	Details of participants	Exclusion criteria	Details of intervention	Details of comparison	Details of all outcomes	Notes
FCM verses ISC								
1.	Jose et al. (15) New Delhi	Open-label RCT (2 arms) with 1:1 allocation ratio Hospital (Tertairy-care) setting Jan 2016 to Aug 2017	Pregnant women diagnosed with moderate to severe IDA (*N* = 100) Mean age (Mean ± SD) FCM: 27.5 ± 3.9 ISC: 26.2 ± 3.6	Anemia due to causes other than IDA Any chronic infections Raised serum transaminases & serum creatinine level Allergic to iron infusion	FCM (*n* = 50 → 50) Max dose of 1 g in 200 mL of 0.9% NS IV infusion for 30 min Subsequent doses (if needed) on day 7 and 14 and were rounded off nearest to 100 mg	ISC (*n* = 50 → 50) ISC infusion 300 mg in 200 mL NS for 15–20 min twice weekly till dosage was completed (<600 mg/ wk)	Hb (g/L) Serum ferritin (μg/L) Serum Iron (μg/dL) TIBC (μg/dL) MCV, MCH, MCHC Transferrin saturation (%) All outcomes assessed at BL, 3,6 & 12 wks	Funding status: NR Cost of drug + consumables in INR FCM: 6872.4 ± 379.7 ISC: 6566.3 ± 449.8 (*p* = 0.0004)
2.	Rathod et al. (28) Cuttack	Double-blind RCT (3 arms) Medical College Sept 2010 to Aug 2012	Post-partum women with IDA (*N* = 366) Mean age (Mean ± SD) FCM: 25.9 ± 3.57 ISC: 26.0 ± 3.66 Oral iron: 25.4 ± 3.05	Blood disorders: SCA, Thalassemia, Aplastic anemia, Megaloblastic anemia Anemia due to liver disease, kidney disease, cardiovascular disease Recent blood transfusion Allergic to parenteral iron	FCM (*n* = 100 → 86) Max single dose of 1 g in 250 mL 0.9% NS as drip infusion over 15 min Not more than one/week Max 0.3 mL FCM injection (15 mg iron/kg body wt.)	ISC (*n* = 100 → 78) ISC according to iron deficit Max dose of 300 mg elemental iron diluted in 300 mL of 0.9% NS as slow IV infusion over 30 min Repeated on alt days when necessary	Hb (g/dL) Serum ferritin (ng/mL) Adverse events Patient satisfaction All outcomes assessed at BL, 2 & 6 wks	Funding status: NR
3.	Naqash et al. (19) Jammu & Kashmir, India	Phase IV RCT (2 arms) (ISRCTN14484575) Medical College and Hospital Duration of study: May 2015 to February 2016	Female patients >18 years with IDA (*N* = 200) Mean Age (Mean ± SD) FCM: 30·41 ± 7·99 ISC: 27·32 ± 4·15 # of participants Pregnancy FCM/ ISC: 48/47 Post-partum FCM/ ISC: 19/20 Menorrhagia FCM/ ISC: 18/20 Others: FCM/ ISC: 15/13	Patients with Uncontrolled HTN Impaired renal, liver function Heart disease	FCM: *n* = 100 → 94 Max single dose of 1 g in 250 mL of 0.9% NS slow infusion for 45 min Subsequent doses on day 8 and 15	ISC: *n* = 100 → 93 Max dose of 200 mg diluted in 200 mL (0.9%) NS slow infusion for 30 min Rest of doses, as and when required were given on alternate days	Hb (g/dL) Serum Iron (μg/dL) Serum ferritin (μg/dL) Transferrin Saturation (%) TIBC (μg/dL) MCV (fL)All outcomes assessed at BL, 2 and 4 wks	Funding status: NR
4.	Mahey et al. (27) New Delhi	Open-label RCT (2 arms) Hospital setting Apr 2013 to May 2014 (CTRI/2015/09/006224)	Anemic patients >18 years with IDA experiencing heavy uterine bleeding (menorrhagia) (*N* = 60) Mean Age (Mean ± SD) FCM: 36.3 ± 9.0 ISC: 35.2 ± 7.5	Anemia with any cause other than IDA Haemochromatosis, chronic infections, gynecological malignancies, or endometrial hyperplasia Receiving myelosuppressive therapy Consuming alcohol or using illicit drugs Raised Sr. transaminase & Sr. creatinine level	FCM: *n* = 30 → 29 Max dose of 1 g in 200 mL of 0.9% NS over 15 min once a week	ISC: *n* = 30 → 29 300 mg in 200 mL 0.9% NS over 2 h twice a week	Hb (g/dL) Serum iron (μg/L) MCV (fL), MCH (pg), MCHC (g/L) All outcomes assessed at BL, 1, 6 and 12 Weeks	Funding status: NR Total per-patient costs in INR FCM: 2860.67 ± 491.8 ISC: 3298.67 ± 357.13 (*p* = 0.001)
5.	Patel et al. (8) Maharashtra	Prospective, RCT (2 arms) Tertiary care hospital May 2016 to April 2018	Antenatal women from 28 to 34 weeks gestation with moderate to severe anemia (*N* = 100) Hb levels: 6–9.9 g% Ferritin <30 ng/mL Age Between 18 to 32 years	Anemia other than IDA Hypersensitive to any iron preparation H/o bleeding tendencies Thalassemia or haemochromatosis Chronic renal failure, CVD, TB, hepatitis B, hepatitis C or HIV infection	FCM: *n* = 50 → 50 1 g of FCM in 200 mL 0.9% NS over 30 min	ISC: *n* = 50 → 50 200 mg in 100 mL 0.9% NS over 30 min on day 0, 2, 4, 6 and 8 (total 1 g)	Hb (gm%) Serum ferritin (ng/mL) Adverse reactions All outcomes assessed at BL and 3 weeks	Funding status: NR Cost of drug in INR FCM: 3310 Rs for 1 g in single dose ISC: 4050Rs fpr 1 g divided in 5 doses
6.	Dakhale et al. (29) Central India	Parallel, open label prospective study (RCT) Tertiary care hospital June 2019 to December 2020	Post-partum women with IDA (*N* = 60) Hb <10 g/dL Mean Age (Mean ± SD) FCM: 24.93 ± 0.59 ISC: 25.13 ± 0.69	Anemia due to other causes as aplastic, megaloblastic or haemolytic anemia Acute or chronic infection Inflammation Liver or renal disease Recent administration of IV iron preparations Blood transfusion Intolerance to iron derivatives	FCM: 30 → 30 500 mg FCM in 100 mL NS at BL	ISC: 30 → 30 200 mg dissolved In 100 mL NS 1st dose: BL 2nd dose: After 2 wks If needed, one additional dose of 100 mg was given	Hb Serum ferritin Adverse reactions All outcomes assessed at BL and 4 wks	Funding status: NR Study drugs were procured from Vinayak agency, Gandhibag, Nagpur and were given free of cost to the patients
7.	Parikh A et al. (20) Gujarat	Prospective comparative randomized analytical study (2 arms) Hospital setting September 2017 to August 2018	Pregnant women of 28–32 weeks gestation with Hb 5 to 9.5 gm% with IDA of pregnancy. (*N* = 100)	Anemia not caused by iron deficiency Hypersensitivity to FCM and IS. Sickle cell disease Not consenting	FCM: *n* = 50 → 50 Max dose 1 g in 200 mL 0.9% NS over 30 min	ISC: *n* = 50 →50 Total 1 g of ISC divided in 5 doses on alternate days (i.e., 200 mg) in 100 mL 0.9% NS over 15–20 min twice a week Not >600 mg/week	Hb (gm/dL) Serum Ferritin (mg/l) Adverse reaction All outcomes assessed at BL, 4 weeks and 90 days after initiation of treatment	Funding status: Funded by Emcure Pharmaceutical, Pune, India In addition 5 mg Folic acid orally once daily were given to the participants
8.	Agrawal and Masand (14) Jaipur, Rajasthan	Prospective RCT (2 arms) Hospital setting August 2018 to January 2019	Pregnant women from gestational age 20 to 36 weeks, Hb <11 gm% and serum ferritin levels <30 ng/mL (*N* = 100) Age between 20–40 yrs	Anaphylaxis to iron substitutes HTN Cardiac, renal, hepatic and endocrine disease Anemia due to chronic disease Worm infestation	FCM: *n* = 50 → 50 IV FCM (1 g/week).	ISC: *n* = 50 →50 IV iron sucrose 200 mg on alternate day, maximum-600 mg/week	Hb gm/dL Serum ferritin ng/mL Adverse reactions All outcomes assessed at BL and 3 weeks post infusion.	Funding status: NR
9.	Patil and Tehalia (21) India	Single-centric, parallel group, open label RCT (3 arms) Tertiary care teaching institute and research center October 2013 to June 2015	Pregnant women 24 to 34 weeks of gestation with Hb between 6.5 g/dL to <9.0 g/dL (N = 150) Age (Mean ± SD) FCM = 25.78 ± 3.68 ISC = 25.66 ± 3.45 ISr = 24.94 ± 3.3	Anemia not linked to iron deficiency, intolerance to iron derivatives H/o asthma, thromboembolism, seizures, drug abuse Renal or hepatic dysfunction.	FCM: *n* = 50→50 Single dose of 1 g in 100 mL NS over 15 min.	ISC: *n* = 50→50 200 mg/day over 20 min in 100 mL NS for 5 days (total 1 g)	Hb (g/dL) RBC count (μ/L) PCV (%) MCH (pg) MCHC (g/dL) MCV (fl) Reticulocyte count (%) Serum Ferritin (μ/L) Adverse reactions All outcomes assessed at BL, 2 and 6 weeks.	Funding status: NR
10.	Khatun and Biswas (18) Kolkata	Double arm, prospective, single center, comparative interventional RCT Medical college and Hospital setting	Pregnant women between 16–34 weeks and single viable fetus with no anomalies Age: 18 year and above IDA with Hb: 7–10 gm% Admitted in antenatal ward	Pregnant women with anemia due to causes other than IDA H/o blood transfusion, erythropoietin treatment, other medical disorders or hematological diseases Allergy to iron derivatives	FCM:n = 90 → 90 1 gm as single dose diluted in 200 mL of 0.9% NS over 30 min	ISC: n = 90 → 90 1gm divided in 4 equal doses on day l, 3, 5, 7 in 100 mL of 0.9% NS given as slow IV infusion over 30 min	Hb (g/dL) Serum ferritin Adverse reaction	
11.	Sharma N et al. (30) North-eastern region	Double arm, prospective comparative study (Non-RCT) Tertiary care health center January 2015 to July 2016	Post-partum patients with Hb < 10 gm/dL. (N = 120) Mean age (Mean ± SD) FCM: 27.38 ± 4.65 ISC: 29.9 ± 5.10	Patients with anemia other than IDA. H/o blood transfusion. H/o allergy to injection iron.	FCM: n = 60 → 60 1 g in 250 mL of NS over 15 min	ISC: n = 60 → 60 200 mg in 200 mL of NS over 20–30 min every alternate day till required dose is completed. Max dose: 600 mg/week	Mean Hb (g%) Serum ferritin Adverse reaction	Funding status: NR
12.	Kaur et al. (17) Haryana	Single arm, prospective study Hospital (Subdistrict) setting Aug 2018 to Feb 2019 CTRI/2018/06/014332	Moderately and severely anemic (Hb: 5.0 and 9.9 g/dL) post-partum women within 48 h of delivery (N = 100) Age Between 18 to 35 yrs	Renal or hepatic impairment, Hb <5 g/dL Allergic to iron formulations H/o parenteral iron or blood transfusion during current pregnancy Any chronic/systemic illness Any blood disorders	FCM: n = 100 → 57 Single dose of FCM in 100 mL of 0.9% NS over 15 min under supervision of a physician within 48 h of delivery	No comparison group	1. Hb (g/dL) by digital hemoglobinometer from capillary blood 2.Serum Ferritin (ng/mL) by enhanced chemiluminescence assay from venous blood All outcomes assessed at BL, 6 wks and 6 mths	Funding status: NR Hb (Mean ± SD) BL: 8.6 ± 1.1 6 Wks: 12.5 ± 1.3 6 Mths: 12.5 ± 1.2 Serum Ferritin BL: 18.7 ± 21.0 6 Wks: 157.7 ± 145.0 6 Mths: 72 ± 52.1
13.	Kant et al. (16) Haryana	Single arm, Open-label trial Hospital (Subdistrict) setting June 2016 to Dec 2016	Pregnant females with a 16 to 32 weeks of gestation with moderate-to-severe anemia attending hospital (N = 60) Age (Mean ± SD) 23.2 ± 3.1	Renal or hepatic impairment Hb <5.0 g/dL H/o parenteral iron administration H/o blood transfusion during current pregnancy Allergic to iron preparations Thalassaemia, SCA or haemolytic anemia	FCM: n = 95→77 First follow up: n = 63 Second follow up: n = 62 Max dose of 1 g FCM in 100 mL of NS over 10–15 min	No comparison group	Hb (g/dL) Serum Ferritin (ng/mL) Adverse events All outcomes assessed at BL, 2 wks (first follow-up) and at delivery (second follow up).	Funding status: NR
FCM verses any other intervention								
14.	Rathod et al. (28) Cuttack	Double-blind RCT (3 arms) Medical College Sept 2010 to Aug 2012	Post-partum women with IDA (N = 366) Mean age (Mean ± SD) FCM: 25.9 ± 3.57 ISC: 26.0 ± 3.66 Oral iron: 25.4 ± 3.05	Blood disorders: SCA, Thalassemia, Aplastic anemia, Megaloblastic anemia Anemia due to liver disease, kidney disease, cardiovascular disease Recent blood transfusion Allergic to parenteral iron	FCM (n = 114→ 100) Max single dose of 1 g in 250 mL 0.9% NS solution over 15 min Not more than one/week	Oral ferrous ascorbate (n = 100→ 70) Details not reported	Hb (g/dL) *p* = 0.003 Serum ferritin (ng/mL) Adverse events Patient satisfaction All outcomes assessed at BL, 2 & 6 wks	Funding status: NR
15.	Damineni et al. (31) Mangalore	Hospital-based prospective randomized comparative study (2 arms) Medical College Sept 2013 to Sept 2015	Women with peripheral smear showing microcytic hypochromic anemia (N = 90) Mean age Oral Iron: 27.4 FCM: 28.04	Anemia other than IDA Receiving myelosuppresive therapy Recent blood transfusions (within 3 months) Therapy with erythropoietin within 3 months prior to screening	FCM: n = 47 → 45 Single dose of 1 g in 250 mL of NS over 15 min	Oral ferrous ascorbate: n = 51 → 45 100 mg BD orally before meals for 6 weeks	Hb (g/dL) Adverse events Outcome assessed at BL, 1, 6 wks	Funding status: NR

IDA, Iron Deficiency Anaemia; NS, Normal Saline; Hb, Haemoglobin; TIBC, Total Iron Binding Capacity; MCV, Mean Corpuscular Volume; BL, Baseline; SCA, Sickle Cell Anaemia.

### 3.1 Details of ongoing studies

Details of four ongoing studies can be found in Supplementary Table S1.

### 3.2 Risk of bias in included studies

**Figure 2:**
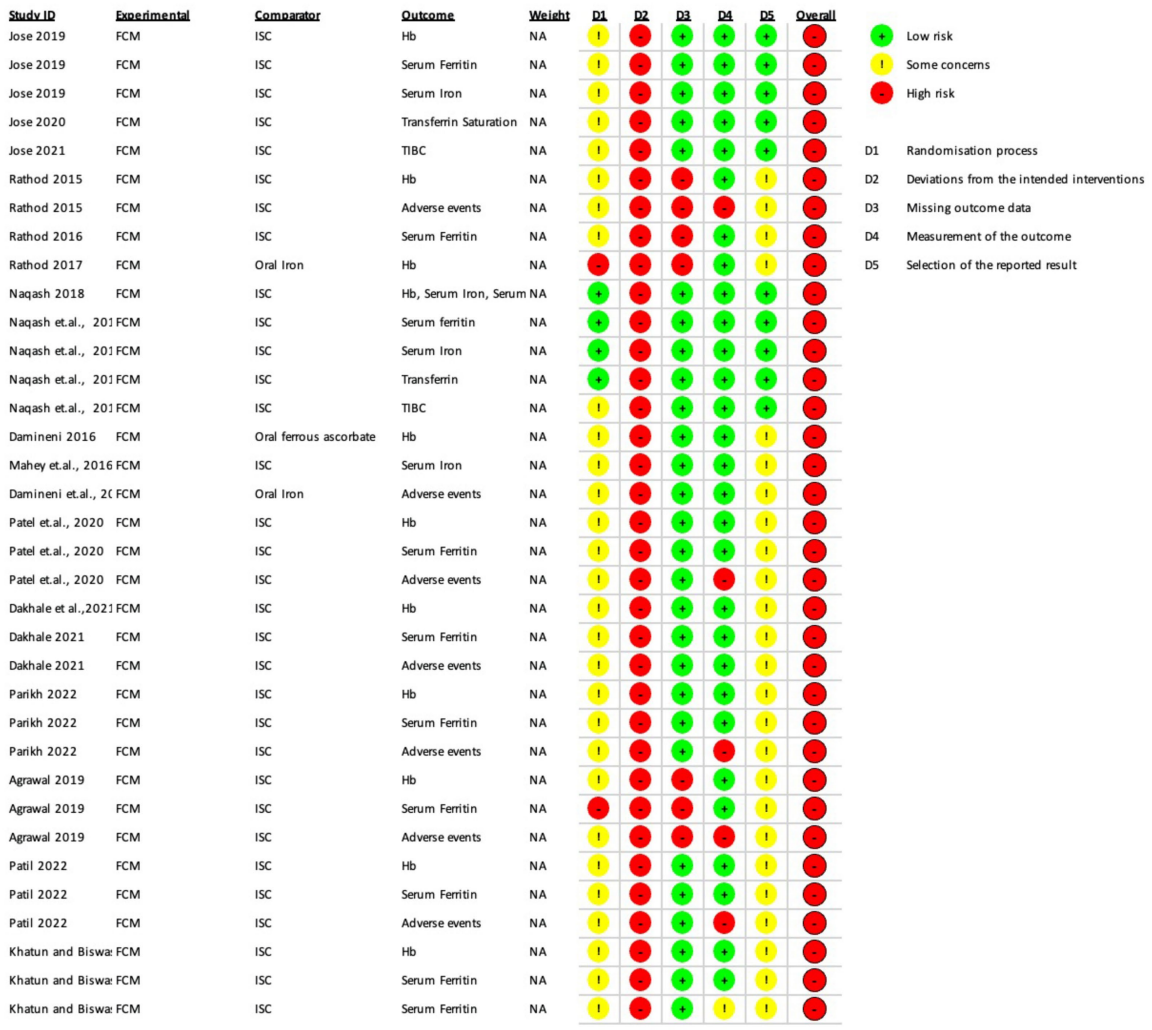
Risk of Bias (RoB 2 tool) assessments in included randomized controlled trials.

**Figure 3:**
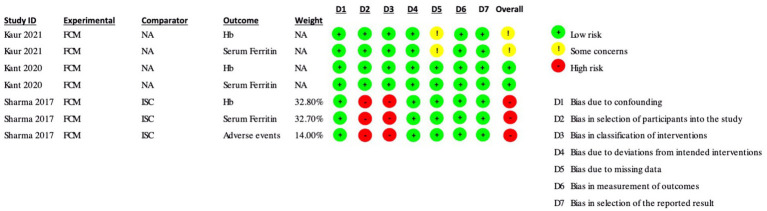
Risk of Bias assessments (ROBINS-I) in included non-randomized controlled trials and single-arm studies.

The Risk of Bias (ROB 2) assessment was conducted for several studies, and the results are summarized below and depicted in Figures 2, 3.

In the study by Jose et al. (15) several outcomes such as hemoglobin, serum ferritin, serum iron, transferrin saturation, and TIBC had some concerns overall.

In Rathod et al. (28) iron sucrose group, both hemoglobin and serum ferritin had high risk of bias overall due to deviations from intended intervention and missing outcome data. Adverse events also had a high risk of bias overall due to the outcome’s measurement, deviations from the intended intervention, and missing outcome data.

Naqash et al. (19) study had a high risk of bias in all outcomes, namely hemoglobin, serum ferritin, serum iron, transferrin saturation, and TIBC, due to deviations from intended interventions. TIBC also had some concerns due to randomization process.

In Damineni et al. (31) there was a high risk of bias in hemoglobin and adverse events outcomes due to the high risk of deviations from intended interventions and some concerns due to the randomization process and selection of reported results in the group where oral ferrous ascorbate was the comparator.

In Mahey et al. (27) there was a high risk of bias in hemoglobin, serum iron, and adverse events due to the high risk of deviations from intended interventions and some concerns due to the randomization process and selection of reported outcome.

In Patel et al. (8) hemoglobin, serum ferritin, and adverse events had an overall high risk of bias due to the high risk of deviations from intended interventions and some concerns due to the randomization process and selection of reported results. Adverse events also had a high risk in measuring the reported outcome.

Dakhale et al. (29) had an overall high risk of bias in hemoglobin, serum ferritin, and adverse events due to the high risk of deviations from intended interventions and some concerns due to the randomization process and selection of reported outcome.

Parikh and Agarwal et al. (20) also had an overall high risk of bias due to the high risk of deviations from intended interventions and some concerns due to the randomization process and selection of reported results. Adverse events also had a high risk in the measurement of reported results.

In Agrawal and Masand et al. (14) there was an overall high risk of bias in hemoglobin, serum ferritin, and adverse events outcomes due to deviations from intended intervention and missing outcome data along with some concerns in the selection of reported results. Hemoglobin also had some concerns in the randomization process, serum ferritin had a high risk in the randomization process, and adverse events had a high risk in the measurement of outcomes.

In Patil and Tehalia et al. (21) iron sucrose comparator group, both hemoglobin and serum ferritin had an overall high risk of bias due to deviations from intended interventions, some concerns due to the randomization process, and selection of reported results. Adverse events outcomes also had high risk in the measurement of the outcome. In the ISC group of Patil et al., hemoglobin had a high risk of bias due to the high risk of deviations from intended interventions and some concern in the randomization process and selection of reported results. In the oral iron group of Patil et al., both adverse events and serum ferritin had a high risk of bias due to the high risk of deviations from intended interventions and some concern in the randomization process and selection of reported outcomes.

In the ROBINS-I assessment, Kaur et al. (17) had only some concerns about the hemoglobin and serum ferritin outcomes due to some concerns of bias due to missing data. Kant et al. had a low risk of bias in both the hemoglobin and serum ferritin outcomes. Sharma et al. (30) had an overall high risk of bias in the hemoglobin, serum ferritin, and adverse events outcomes due to a high risk of bias in selecting participants for the study and bias in the classification of interventions.

To summarize, the studies reviewed in the ROB 2 tool had various levels of risk of bias in different outcomes, with some studies having a high risk of bias in multiple outcomes and others having only some concerns in one or two outcomes. The ROBINS-I tool assessed the risk of bias differently, with some studies having a low risk of bias in certain outcomes and others having a high risk of bias overall. Both tools provided a systematic approach to evaluating the risk of bias in studies, which was crucial for accurately evaluating their outcome.

### 3.3 Effects of interventions

#### 3.3.1 Comparison 1: ferric carboxymaltose versus iron sucrose complex

##### 3.3.1.1 Anemia

None of the included studies reported this outcome.

##### 3.3.1.2 Hemoglobin

**Figure 4:**
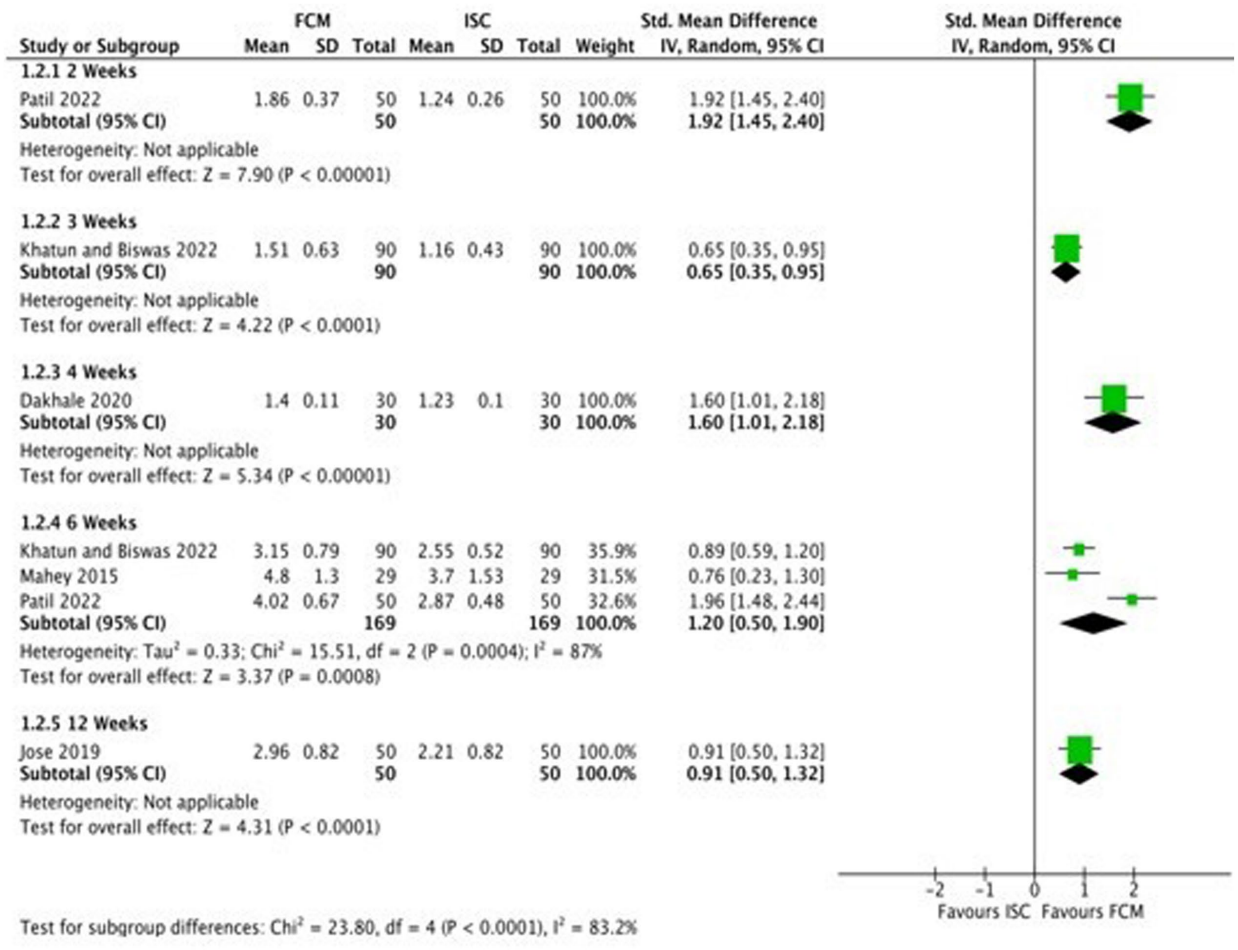
Forest plot of comparison: 1 FCM verses ISC, outcome: hemoglobin (change-scores).

Six studies compared FCM with ISC on hemoglobin levels in moderate to severe anemic participants as post-scores, and four studies as change-scores. The outcomes were assessed at 2, 3, 4, 6 and 12 weeks. At 12 weeks, all studies showed significant improvements in Hb levels at post-scores and change-scores except for Mahey et al. (27) (Figure 4; Supplementary Figure S1).

##### 3.3.1.3 Adverse events

**Figure 5:**
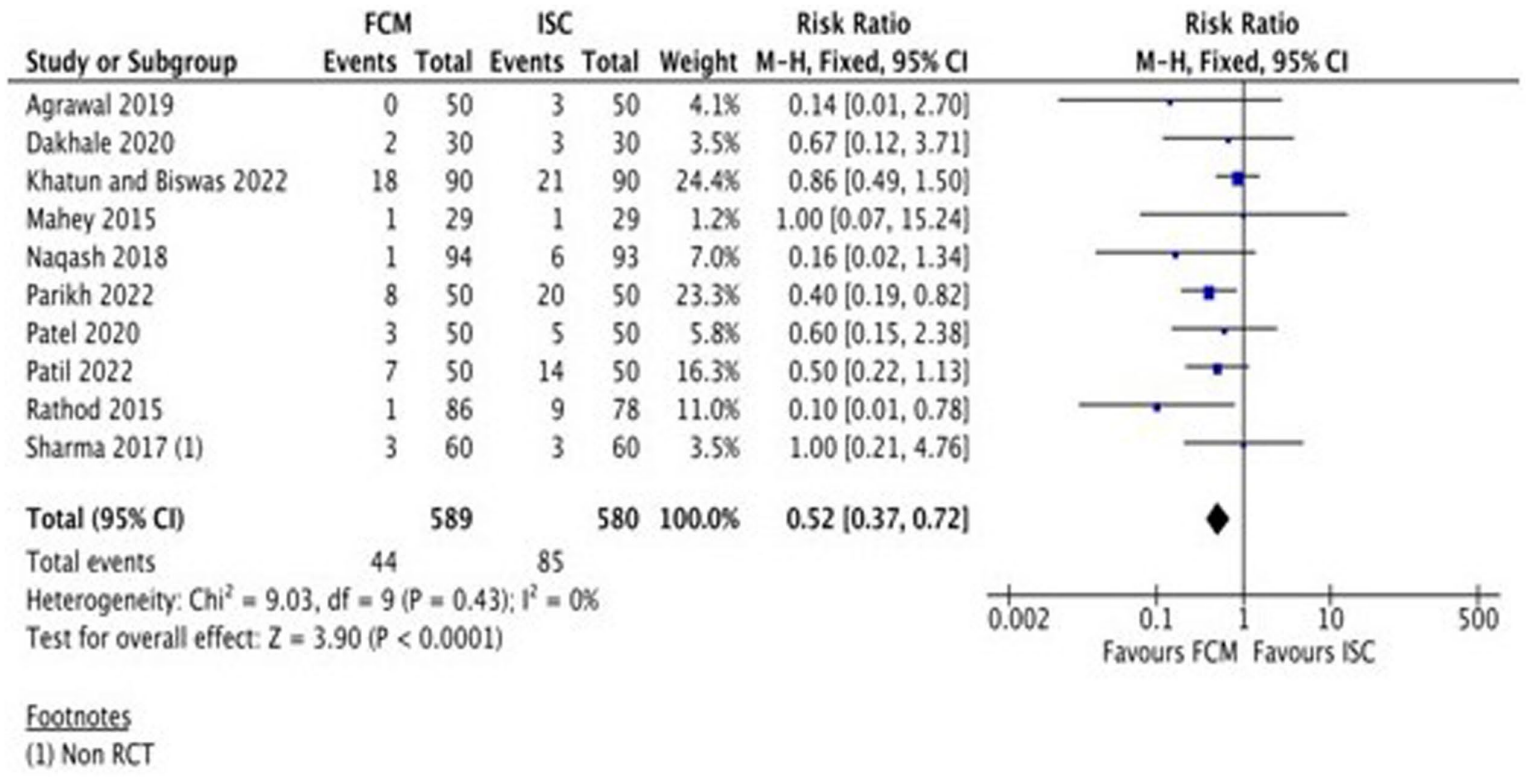
Forest plot of comparison: 1 FCM verses ISC, outcome: adverse events.

A total of eight studies (Table 2) assessed the adverse events of FCM and ISC when administered in moderate to severely anemic participants. All studies showed fewer adverse events with FCM as compared to ISC. The pooled analysis shows that the risk of adverse events in FCM group was 48% less than in ISC group (RR 0.52, 95% CI 0.37 to 0.72; participants = 1,169; studies = 10; I^2^ = 0%) (Figure 5; Supplementary Figure S2).

**Table 2:** Adverse events.

Study ID	FCM	ISC
Agrawal and Masand (14)	No adverse events	Skin rashes Systemic reactions (fever, chills, breathlessness, rashes)
Dakhale et al. (29)	Pain at injection site (2/30) Headache (1/30) Nausea (1/30)	Pain at injection site (3/30) Headache (1/30) Nausea (1/30)
Khatun and Biswas (18)	Swelling at injection site (4/90) Nausea (2/90) Nausea, vomiting (5/90) Pruritus (2/90) Redness on injection site (5/90)	Swelling at injection site (7/90) Nausea (5/90) Nausea, vomiting (6/90) Muscle cramp (3/90)
Mahey et al. (27)	Itching and a rash (1/30) within 15 min that subsided within 30 min	Fever (39.4°C) and vomiting within 6 h that improved within 24 h
Naqash et al. (19)	Mild headache after 2nd dose (1/94)	Arthralgia at 7th dose (1/93). Nausea (3/93) Tingling sensation (3/93) Headache after 6th dose (2/93)
Parikh and Agarwal (20)	Pain/burning at injection site (4/50) Swelling at injection site (2/50) Blackening at injection site (0/50) Nausea/vomiting (0/50) Gastritis (2/50) Giddiness/hypotension (0/50) Other (0/50)	Pain/burning at injection site (9/50) Swelling at injection site (4/50) Blackening at injection site (0/50) Nausea/vomiting (4/50) Gastritis (1/50) Giddiness/hypotension (2/50) Other (0/50)
Patel et al. (8)	Mild local reaction (3/50)	Mild local reaction (4/50) Severe anaphylactic reaction (1/50)
Patil et al. (21)	Shivering Local phlebitis (4/50) Headache	Shivering Local phlebitis (7/50) Headache
Rathod et al. (28)	Arthralgia, tingling sensation and headache (1/86)	Joint pain and tingling sensation (6/78) Transient hypotension (3/78)
Sharma et al. (30)	Burning at injection site (1/60) Headache (1/60) Fever (1/60)	Burning at injection site (1/60) Headache (1/60) Tingling sensation (1/60)

None of the studies reported any serious adverse drug reaction in FCM group requiring hospitalization.

##### 3.3.1.4 Serum ferritin

**Figure 6:**
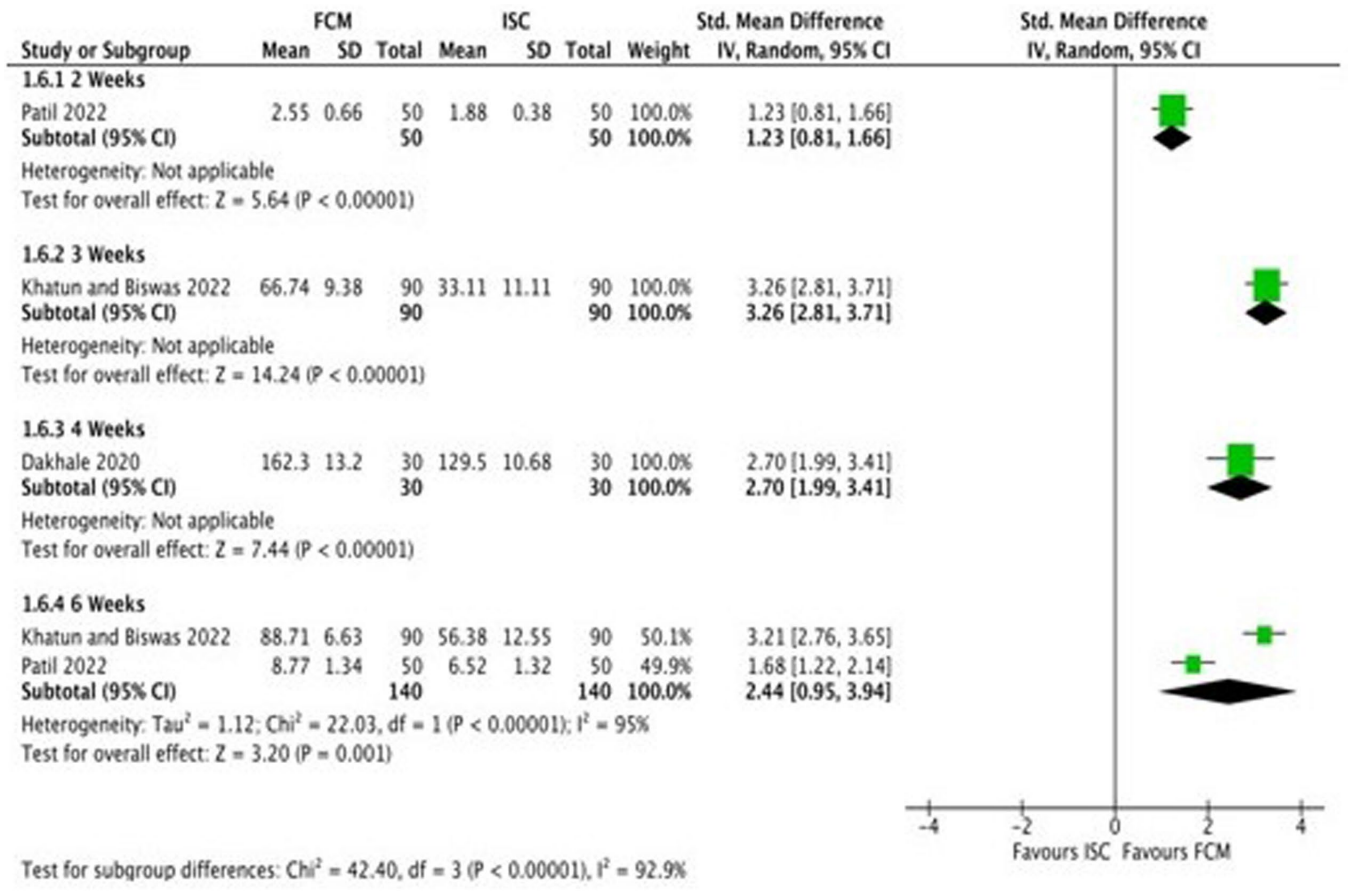
Forest plot of comparison: 1 FCM verses ISC, outcome: serum ferritin (change-scores).

A total of six studies (18–21, 28, 30) reported data on serum ferritin levels at baseline and at the end of the treatment. Studies reported serum ferritin levels at baseline and at different time points after 2, 3, 4, 6, and 12 weeks (18–21, 28, 30). Three studies reported change scores of serum ferritin levels (18, 21, 29). Subgroup analysis was undertaken according to the different time points. All the included studies demonstrate that the serum ferritin levels in the FCM group were significantly higher than in the ISC group. As the heterogeneity amongst the studies was substantial (I^2^ = 98.3% for post-scores and I^2^ = 92.9% for change-scores), and as some studies (18, 21, 28) reported serum ferritin levels at different time points, we did not pool the findings of the studies (Figure 6; Supplementary Figure S3).

##### 3.3.1.5 Serum iron

Only two studies (19, 27) reported data on serum iron levels before treatment and after treatment at 4 weeks, 6 weeks, and at 12 weeks. One study by Mahey et al. (27) reported data at 6, and 12 weeks and Naqash et al. (19) at 4 weeks. Subgroup analysis was undertaken according to the different time points. All the included studies demonstrate that the serum ferritin levels in the FCM group were higher than in the ISC group. As the heterogeneity amongst the studies was substantial (I^2^ = 99.2%), and as one study (27) reported serum ferritin levels at different time points, we did not pool the findings of the studies (Supplementary Figure S4).

##### 3.3.1.6 Total iron binding capacity (TIBC)

Only two studies (15, 19) reported data on TIBC. One study by Jose et al. (15) reported data at 3, 6, and 12 weeks and Naqash et al. (15), at 4 weeks. Subgroup analysis was undertaken according to the different time points. All the included studies demonstrates that the serum iron levels in the FCM group were lower than in the ISC group in two studies at 3 weeks and at 4 weeks (19) but not at 6 and 12 weeks (15). As the heterogeneity amongst the studies was substantial (I^2^ = 99.2%), and as one study (15) reported serum ferritin levels at different time points, we did not pool the findings of the studies (Supplementary Figure S5).

#### 3.3.2 Comparison 2: ferric carboxymaltose versus iron sorbitol

##### 3.3.2.1 Anemia

None of the included studies reported this outcome.

##### 3.3.2.2 Hemoglobin

**Figure 7:**
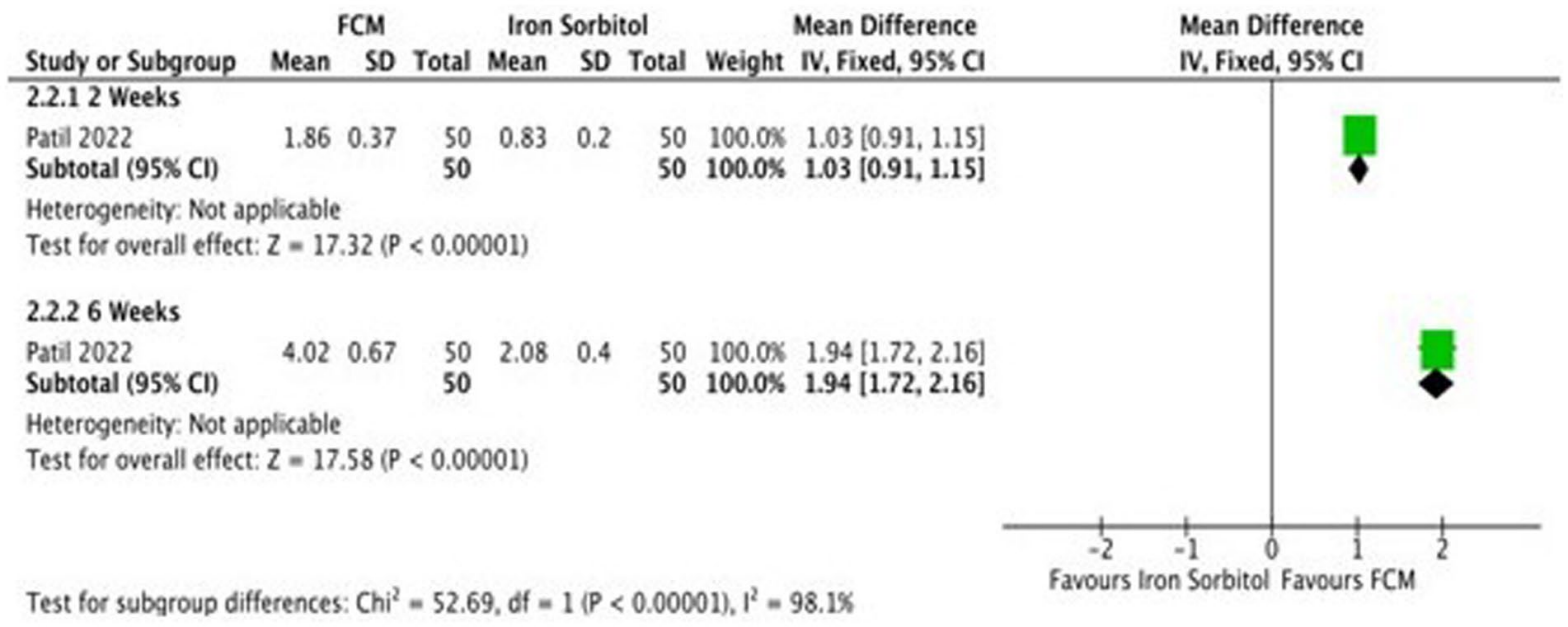
Forest plot of comparison: FCM verses alternative experimental treatment modality, outcome: hemoglobin (change-scores).

Only one study (21) compared FCM with intramuscular injection of iron sorbitol on hemoglobin levels in anemic participants as post-scores and two studies as change-scores. The outcomes were assessed at 2 and 6 weeks. The study showed significant improvements in Hb levels at post-scores as well as change-scores (Figure 7; Supplementary Figure S6).

##### 3.3.2.3 Adverse events

Only one study (21) assessed the adverse events of FCM and iron sorbitol when administered to anemic participants. The study showed fewer adverse events with FCM as compared to iron sorbitol. The pooled analysis shows that the risk of adverse events in FCM group was 78% less than that in iron sorbitol (RR 0.22, 95% CI 0.11 to 0.45; participants = 100; studies = 1).

##### 3.3.2.4 Serum ferritin

**Figure 8:**
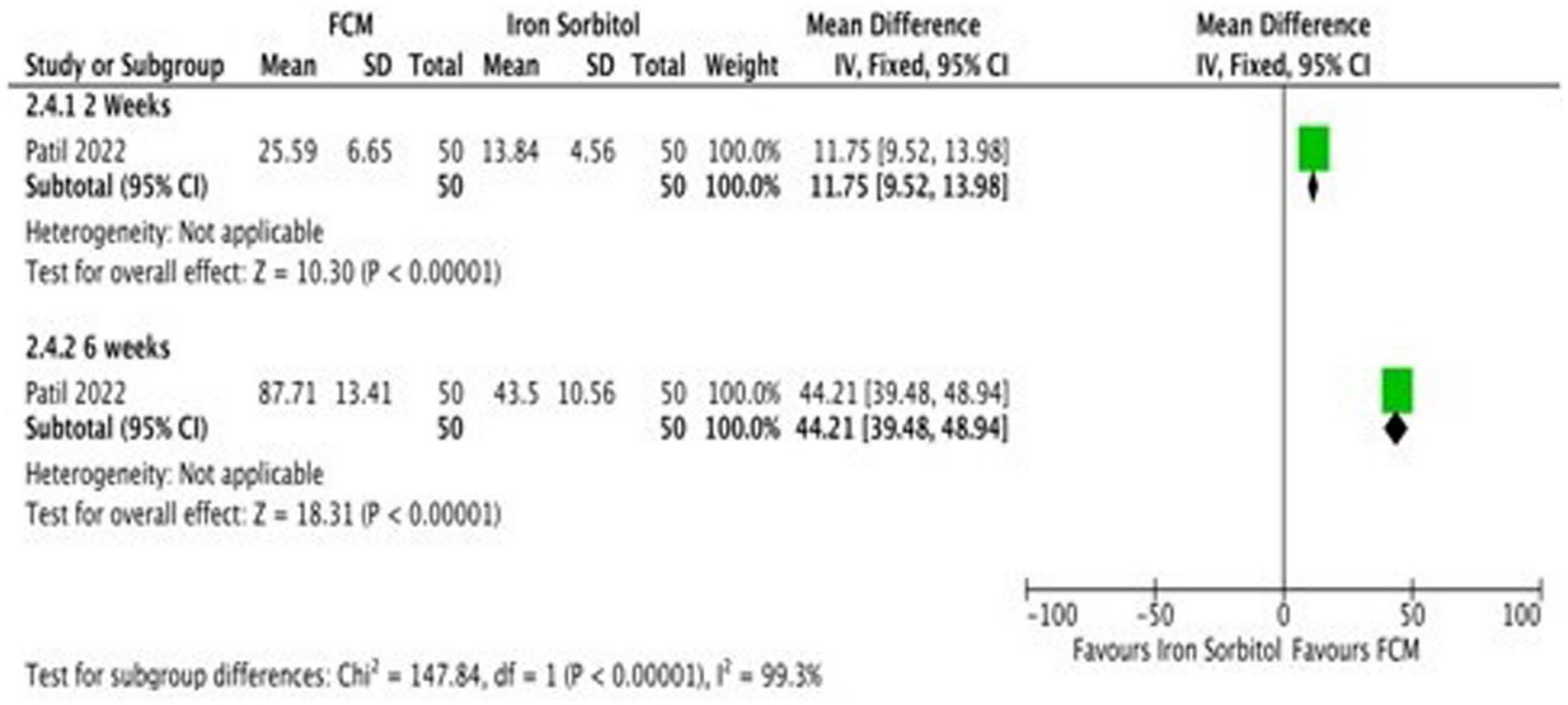
Forest plot of comparison: FCM verses alternative experimental treatment modality, outcome: serum ferritin (change scores).

Only one study (21) detailed the data on serum ferritin levels at baseline and at the end of the treatment and changed scores at baseline level and at 2 weeks, and 6 weeks. Subgroup analysis was undertaken according to the different time points. The study demonstrates that the serum ferritin levels in the FCM group were significantly higher as compared to iron sorbitol. The serum ferritin levels were higher at 6 weeks as compared to 2 weeks (Figure 8; Supplementary Figure S7).

##### 3.3.2.5 TIBC

None of the included studies reported this outcome.

#### 3.3.3 Comparison 3: ferric carboxymaltose versus oral iron

##### 3.3.3.1 Anemia

None of the included studies reported this outcome.

##### 3.3.3.2 Hemoglobin

**Figure 9:**
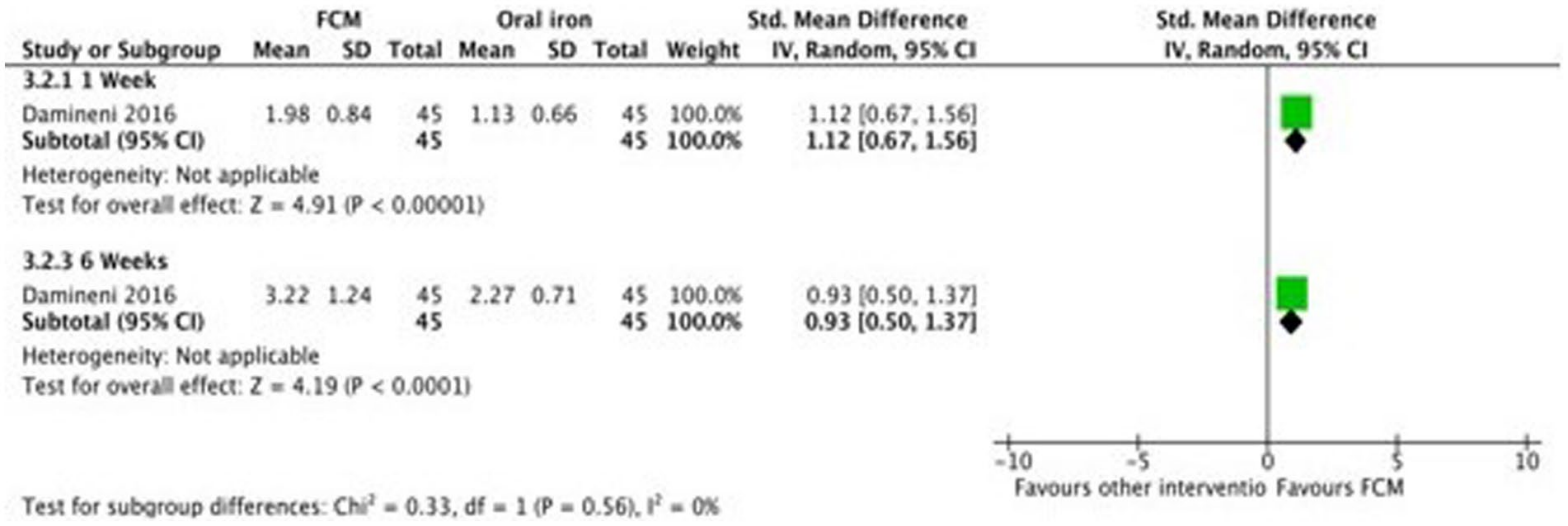
Forest plot of comparison: FCM verses alternative experimental treatment modality, outcome: hemoglobin (change-scores).

Only two studies (28, 31) compared FCM with oral iron (ferrous ascorbate) on hemoglobin levels in anemic participants as post-scores as well as change-scores. In one study (31) outcomes were assessed at 1 week and 4 weeks, and in the other study (28), the outcomes was assessed at 2 weeks and 6 weeks. Both the studies showed significant improvements in Hb levels at post-scores as well as change-scores in FCM group as compared to oral iron (Figure 9; Supplementary Figure S8).

##### 3.3.3.3 Adverse events

**Figure 10:**
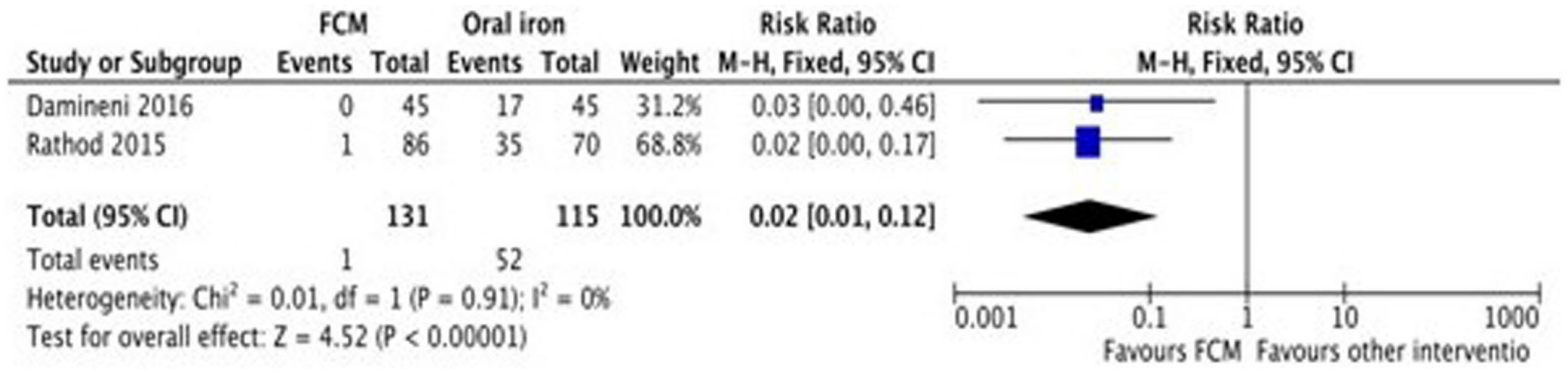
Forest plot of comparison: FCM verses alternative experimental treatment modality, outcome: adverse events.

Only two studies (28, 31) assessed the adverse events of FCM and oral iron (ferrous ascorbate) when administered in anemic participants. The study showed fewer adverse events with FCM as compared to oral iron. The pooled analysis shows that the risk of adverse events in FCM group was 98% less than in oral iron (RR 0.02, 95% CI 0.01 to 0.12; participants = 246; studies = 2) (Figure 10).

##### 3.3.3.4 Serum ferritin

Only one study (28) reported data on serum ferritin levels at baseline and at the end of the treatment at 2 weeks, and 6 weeks. The study demonstrates that the serum ferritin levels in the FCM group were significantly higher as compared to oral iron. The serum ferritin levels were lesser at 6 weeks as compared to 2 weeks (Supplementary Figure S9).

##### 3.3.3.5 TIBC

None of the included studies reported this outcome.

#### 3.3.4 Comparison 4: ferric carboxymaltose in combination with other treatments versus ferric carboxymaltose treatment alone

None of the included studies assessed this comparison.

### 3.4 Quality of evidence

#### 3.4.1 GRADE assessments for FCM compared to ISC for anemia in Indians

The assessment of hemoglobin quality using Tables 3, 4 revealed ‘low quality’ at 2, 4, and 6 weeks, “moderate quality” at 3 weeks, and “very low quality” at 12 weeks. This was attributed to a high risk of bias and limited participant numbers in the analysis. The quality of evidence for serum ferritin was “low quality” at 2, 4, and 12 weeks, and “very low quality” at 6 weeks due to factors like high risk of bias, presence of heterogeneity, and a few number of participants in the analysis. The quality of evidence for adverse events was assessed as ‘moderate quality’ due to the presence of high risk of bias.

**Table 3:** GRADE assessments for ferric carboxymaltose compared to iron sucrose complex (ISC) for anemia in Indians.

**FCM compared to ISC for anemia in Indians**					
**Patient or population:** Anaemic Indians **Setting:** Hospital Settings **Intervention:** FCM **Comparison:** ISC					
**Outcomes**	**No of participants** **(studies)** **Follow-up**	**Certainty of the evidence** **(GRADE)**	**Relative effect** **(95% CI)**	**Anticipated absolute effects**	
				**Risk with ISC**	**Risk difference with FCM**
Hb (g/dL) (Post-scores) – 4 Weeks	187 (1 RCT)	⨁⨁◯◯ Low^b,c^	–	–	SMD**2.48 higher** (2.09 higher to 2.86 higher)
Hb (g/dL) (Post-scores) – 6 Weeks	422 (4 RCTs)	⨁⨁◯◯ Low^b,d^	–	–	SMD**1.04 higher** (0.71 higher to 1.37 higher)
Hb (g/dL)(Post-scores) – 12 Weeks	258 (3 RCTs)	⨁◯◯◯ Very low^b,e,f^	–	–	SMD**0.95 higher** (0.29 higher to 1.6 higher)
Serum Ferritin (μg/dL) (Post scores) – 4 Weeks	187 (1 RCT)	⨁⨁◯◯ Low^a,b^	–	–	SMD**14.33 higher** (12.84 higher to 15.83 higher)
Serum Ferritin (μg/dL) (Post scores) – 6 Weeks	264 (2 RCTs)	⨁◯◯◯ Very low^b,g,h^	–	–	SMD**1.21 higher** (0.25 higher to 2.17 higher)
Serum Ferritin (μg/dL) (Post scores) – 12 Weeks	100 (1 RCT)	⨁⨁◯◯ Low^a,b^	–	–	SMD**2.43 higher** (1.91 higher to 2.95 higher)
Adverse reaction	869 (8 RCTs)	⨁⨁⨁◯ Moderate^g^	not estimable	142 per 1,000	**142 fewer per 1,000** (142 fewer to 142 fewer)
*The risk in the intervention group (and its 95% confidence interval) is based on the assumed risk in the comparison group and the**relative effect** of the intervention (and its 95% CI). **CI:** confidence interval;**SMD:** standardised mean difference					
**GRADE Working Group grades of evidence** **High certainty:** we are very confident that the true effect lies close to that of the estimate of the effect. **Moderate certainty:** we are moderately confident in the effect estimate: the true effect is likely to be close to the estimate of the effect, but there is a possibility that it is substantially different. **Low certainty:** our confidence in the effect estimate is limited: the true effect may be substantially different from the estimate of the effect. **Very low certainty:** we have very little confidence in the effect estimate: the true effect is likely to be substantially different from the estimate of effect.					
**Explanations** a. Downgraded by one for limitation in study design (high RoB) b. Downgraded by one as sample size not optimal c. Downgraded by one for limitation in study design (high RoB in D2 domain of RoB 2) d. Downgraded by one for limitation in study design (high RoB) in three out of four studies e. Downgraded by one for limitation in study design (high RoB) in two out of three studies f. Downgraded by one for inconsistency (heterogeneity) I^2^=84% g. Downgraded by one for limitation in study design (high RoB) in all studies h. Downgraded by one for inconsistency (heterogeneity) I^2^=92%					

**Table 4:** GRADE assessments for ferric carboxymaltose compared to iron sorbitol for anemia in Indians.

**FCM compared to Iron Sorbitol for Anaemia in Indians**					
**Patient or population:** Anaemic Indians **Setting:** Hospital **Intervention:** FCM **Comparison:** Iron Sorbitol					
**Outcomes**	**No of participants** **(studies)** **Follow-up**	**Certainty of the evidence** **(GRADE)**	**Relative effect** **(95% CI)**	**Anticipated absolute effects**	
				**Risk with Iron Sorbitol**	**Risk difference with FCM**
Hb (g/dL)(Change scores) – 2 Weeks	100 (1 RCT)	⨁⨁◯◯ Low^a,b^	–	The mean hb (g/dL)(Change scores)—2 Weeks was**0**	MD**1.03 higher** (0.91 higher to 1.15 higher)
Hb (g/dL)(Change scores)—6 Weeks	100 (1 RCT)	⨁⨁◯◯ Low^a,b^	–	The mean hb (g/dL)(Change scores)—6 Weeks was**0**	MD**1.94 higher** (1.72 higher to 2.16 higher)
Serum Ferritin (μg/l)(Change scores) – 2 Weeks	100 (1 RCT)	⨁⨁◯◯ Low^a,b^	–	The mean serum Ferritin (μg/l)(Change scores)—2 Weeks was**0**	MD**11.75 higher** (9.52 higher to 13.98 higher)
Serum Ferritin (μg/l)(Change scores) – 6 weeks	100 (1 RCT)	⨁⨁◯◯ Low^a,b^	–	The mean serum Ferritin (μg/l)(Change scores)—6 weeks was**0**	MD**44.21 higher** (39.48 higher to 48.94 higher)
Adverse Events	100 (1 RCT)	⨁⨁◯◯ Low^a,b^	**RR 0.22** (0.11 to 0.45)	640 per 1,000	**499 fewer per 1,000** (570 fewer to 352 fewer)
*The risk in the intervention group (and its 95% confidence interval) is based on the assumed risk in the comparison group and the**relative effect** of the intervention (and its 95% CI). **CI:** confidence interval;**MD:** mean difference;**RR:** risk ratio					
**GRADE Working Group grades of evidence** **High certainty:** we are very confident that the true effect lies close to that of the estimate of the effect. **Moderate certainty:** we are moderately confident in the effect estimate: the true effect is likely to be close to the estimate of the effect, but there is a possibility that it is substantially different. **Low certainty:** our confidence in the effect estimate is limited: the true effect may be substantially different from the estimate of the effect. **Very low certainty:** we have very little confidence in the effect estimate: the true effect is likely to be substantially different from the estimate of effect.					
***Explanations*** a. Downgraded by one for limitation in study design (High RoB) b. Downgraded by one for imprecision (small sample size)					

When using the “RoB 2” tool, we found that all included studies had high risk of bias in at least one of the six domains. Most of the included studies did not describe the method of randomization and allocation concealment that may pose a serious selection bias (D1: Randomization process). The majority of the studies were open-label, and it was uncertain if blinding was successful in blinded studies, raising the potential of performance bias (D2: Deviation from intended intervention). Clinical outcome measures, such as hemoglobin, serum ferritin not affected by the subjectivity of the participants, were highly subjected to selection and performance bias whereas adverse events were highly subjected to selection (D1: Randomization process), performance (D2: Deviation from intended intervention), and detection bias (D4: Measurement of the outcome).

The inconsistency was only high for two outcomes (Hemoglobin and serum ferritin, Comparison 1 and 2) owing to a considerable level of heterogeneity, which was addressed through subgroup analyzes. The evidence in this review did not have issues regarding indirectness. Imprecision was an issue owing to small sample sizes, which lowered our confidence in the effects by one level. Except for two studies (15, 19), we did not find the protocol available. Hence, the risk of reporting bias had some concern. There were insufficient trials included in the meta-analyzes to utilize a funnel plot and assess the possible risk of publication bias.

## 4 Discussion

Our findings indicate that FCM can serve as a viable option for women with IDA, addressing not only the correction of hemoglobin deficiency but also the replenishment of iron stores. Other treatment of IDA, such as Iron sucrose complex, Iron sorbitol, or ferrous ascorbate, showed an increase in the hemoglobin level; however, the increment was significantly higher in the participants treated with FCM as compared to ISC infusion or iron sorbitol infusion, or oral iron. Serum ferritin level was also increased in the other treatment modalities but was higher in participants treated with FCM. FCM was well tolerated in patients with IDA, and most drug-related adverse events considered mild to moderate in severity.

The convenient dosing with a lesser total number of required doses decreased the reduces the frequency of hospital visits and, in turn, resulted in satisfactory compliance (18), higher patient satisfaction (28), higher acceptability, better general well-being (28), better HRQOL (19), minimum requirement of hospital resources and increase in acceptability as compared to patients treated with other treatment modalities (19).

### 4.1 Overall completeness and applicability of evidence

Evidence regarding FCM for anemia in India is limited, with data available only from small sample-sized RCTs that limits us from reaching reliable conclusions regarding the effects of FCM. These studies are also limited in their generalisability, as all the studies included women between 18 to 40 years of age.

The results of this systematic review can only be interpreted in consideration of the following factors.

- None of the studies assessed the comparison between FCM in combination with other treatments versus FCM alone.
- Only two studies assessed serum iron and TIBC in FCM and ISC group.
- Only one study assessed hemoglobin and adverse events in FCM verses iron Sorbitol
- Two ongoing studies (Table 2) will substantially increase the amount of available data to analyze.

### 4.2 Potential biases in the review process

Our review followed the principles outlined in Cochrane’s Handbook of Systematic Reviews (32). We executed a thorough search and searched data sources (including multiple databases, and clinical trial registries) that necessitate the inclusion of all published studies concerning FCM formulations. Although language limitations were taken into account, our focus remained on studies published in languages we anticipated. The evaluation of each study’s relevance was carried out meticulously, with the screening process executed by independent reviewers in duplicate. For robustness, data extraction, encompassing assessments of risk of bias (RoB) as well as GRADE assessments, were undertaken in duplicate by two independent reviewers. This dual approach served to guarantee the precision and accuracy of data extraction and reporting.

The present study was based on comprehensive bibliographical search that encompassed the inclusion of all published clinical trials addressing various intravenous formulations.

Due to a lack of details in the methodology and results section of the included studies, we had to pool some incompatible data. Some data were provided graphically in the published reports. The absence of crucial information like standard deviations hindered the execution of specific analyzes.

### 4.3 Agreements and disagreements with other studies or reviews

The findings of this systematic review confirm the results from already published systematic reviews on studies from other countries. In all the available systematic reviews (Table 5), among the different iron formulations available for the treatment of IDA, FCM was found to be superior when compared with other iron regimens in terms of improving hemoglobin levels and serum ferritin levels in different populations with iron deficiency anemia and indicated a high safety profile.

**Table 5:** Findings of other systematic reviews for all countries on effect of FCM for treatment of anemia.

Study ID	Population	Intervention	Comparison	Hemoglobin	Serum Ferritin	Adverse Events	Additional comment
Govindappagari et al. (33)	Pregnant women with IDA Included 11 RCTs from LMICs, HICs	IV Iron (Iron sucrose, FCM, iron dextran)	Oral Iron (Ferrous sulphate, ferrous fumarate, Iron polymaltose complex, ferrous ascorbate)	Achieved target Hb more often OR 2.66 (95% CI: 1.71–4.15; *p* < 0.001; I ^2^ = 47% Increased Hb level after 4 weeks WMD 0.84; 95% CI: 0.59–1.09; p < 0.001; I ^2^ = 89%)	NR	Decreased adverse reactions, OR 0.35 (95% CI: 0.18–0.67; *p* = 0.001; I ^2^ = 74%)	IV iron is superior to oral iron for treatment of IDA in pregnancy Women receiving IV iron more often achieve desired Hb targets, faster and with fewer side effects
Pollock et al. (34)	Patients with IDA	Iron isomaltose (IIM) 5 RCTs of IIM (4 versus oral iron and 1 versus ISC)	FCM 14 RCTs of FCM (11 versus oral iron and 3 versus ISC)	IIM: Significantly larger increase from BL Hb: MD = +0.249 g/dL with IIM relative to FCM	NR	Hypersensitivity Hypophosphatemia	This SR identified no completed RCTs of IIM versus FCM Higher increase from baseline Hb in IIM than FCM
Qassim et al. (35)	Pregnant women with IDA Included 21 RCTs & 26 observational studies from LMICs & HICs	IV iron FCM (4 studies, n = 276) IPM (3 studies, *n* = 164) ISC (41 studies, *n* = 2,635)	Regardless of comparator	All IV iron preparations led to significant improvements in Hb, with a median increase of 2.18 g/dL at 3 to 4 weeks and 3.43 g/dL by delivery Increase in Hb with high dose: 2.5 (2.0–3.96) g/dL Increase in Hb with low dose: 2.0 (6.2–50.3) g/dL	All IV iron preparations led to significant improvements in Ferritin by a median of 27 μg/L over first 4 weeks	Median prevalence of ADR for: IPM: 2.2 (0–4.5) % FCM: 5.0 (0–20% ISC: 6.7 (0–19.5) %	No single preparation of IV iron appeared to be superior
Rognoni et al. (36)	>18 years with IDA Included 21 RCTs from LMICs, HICs	FCM	Other iron formulations (ferric gluconate, oral iron) and placebo	1. FCM vs. ferric gluconate (g/dL)(Change score): MD = 0.6; 95% CI 0.2–0.9 2. FCM vs. oral iron: (Change score): MD = 0.8; 95% CI 0.6–0.9 3. FCM vs. Placebo: (Change score): MD = 2.1; 95% CI 1.2–3.0	FCM verses ferric gluconate(μg/l) (Change score): MD = 1.5; 95% CI 131.4 to 122.8 FCM vs. Oral iron (μg/l)(Change score): MD = 172.8; 95% CI 66.7–234.4 ISC verses FCM (μg/l) (Change score): MD = 21.4; 95% CI 160.7 to 118.4	FCM was well tolerated and associated with a minimal risk of AEs	All currently available IV iron preparations appear to be safe and effective FCM is better with quicker correction of Hb and serum ferritin levels in patients with IDA
Rogozińska et al. (37)	Pregnant women with IDA Included RCTs from LICs, LMICs and HICs	Iron preparations, with at least 60 mg of elemental iron (ISC and FCM)	Another iron or non-iron preparation (Oral ferrous sulfate)	IS verses oral iron: Change-score: MD = 0.71; 95% CI 0.262–1.17 g/dL; 7 trials FCM verses oral iron: Change-score: MD = 0.85; 95% CI 0.051–1.65 g/dL; 1 trial 53 trials (9,145 women), 30 (15 interventions; 3,243 women)	IS: MD 49·66; 95% CI 13·63–85·69 μg/L; 4 trials 15 (9 interventions; 1,396 women)	Less common AE in IS and FCM: local pain, skin irritation, rare occasions, allergic reactions Common AE in oral iron: GI effects (nausea, vomiting, and altered bowel movements)	Good evidence of benefit for ISC and some evidence for FCM
Shin et al. (38)	Obstetric and gynaecologic patients with IDA Included 9 RCTs with 910 patients (FCM: n = 456; ISC: n = 454) from LMICs and HIC	FCM	ISC	Higher Hb in FCM vs. ISC: MD = 0.67; 95% CI 0.25–1.08 g/dL; *p* = 0.002, I^2^ = 92%	Higher in FCM vs. ISC: MD = 24.41; 95% CI, 12.06–36.76; *p* = 0.0001; I^2^ = 75%	Lower incidence of AE in FCM than ISC: RR, 0.53; 95% CI 0.35–0.80; *p* = 0.003; I^2^ = 0%	FCM group showed better efficacy in increasing Hb and ferritin levels and a favorable safety profile with fewer adverse events compared with IS group

OR, Odds Ratio; ISC, Iron sucrose complex; BL, Baseline; MD, Mean Difference; Iron polymaltose (IPM); LMICs, Low Middle-Income Countries; HICs, High-Income Countries; RR, Risk Ratio.

## 5 Conclusion

The evidence from this SR does not support a robust clinical efficacy conclusion. In summary, this review indicates that a gradual single 1 g FCM infusion is both safe and effective for treating IDA in women. FCM demonstrates superior elevation of hemoglobin levels and restoration of iron stores compared to other interventions (ISC, Iron sucrose, and ferrous ascorbate), with minimal adverse events. However, the evidence is of ‘low’ to ‘very low’ quality, primarily based on ten studies with a high risk of bias and insufficient participant numbers for conclusive results. Further research is likely to influence these findings. FCM consistently shows fewer adverse events than other interventions, with evidence ranging from ‘moderate’ to ‘very low’ quality. The outcomes indicate FCM’s well-tolerated, safe, and effective role as an alternative to other interventions for IDA in women.

FCM offers the advantage of swiftly addressing IDA in some patients within just 2 weeks, requiring a single dose without the need for repeated administrations—thus offering a more convenient treatment approach. This approach also offers benefits such as administering a substantial dose per session, minimizing the number of required doses, reducing hospital visits, lowering transportation costs, needing less infusion-related equipment, and alleviating patient discomfort linked to multiple needle insertions.

However, despite these advantages, the body of evidence in this SR falls short of supporting a definitive conclusion regarding clinical efficacy.

### 5.1 Implications for research

Information from adequately powered, multicentric, methodologically rigorous RCTs to compare the efficacy and safety of FCM over other alternative treatment modalities for the treatment of anemia are necessitated. Cost-effectiveness analyzes are also necessitated.

## Data availability statement

The original contributions presented in the study are included in the article/Supplementary material, further inquiries can be directed to the corresponding author.

## Author contributions

MK: Supervision, Writing – review & editing, Conceptualization, Funding acquisition, Formal analysis, Investigation, Methodology, Software, Validation, Writing – original draft. AS: Conceptualization, Formal analysis, Investigation, Resources, Validation, Supervision, Writing – review & editing. SG: Conceptualization, Validation, Visualization, Writing – review & editing. SU: Data curation, Formal analysis, Methodology, Software, Writing – original draft, Writing – review & editing. NW: Data curation, Formal analysis, Methodology, Writing – original draft. AA: Data curation, Formal analysis, Methodology, Software, Writing – original draft, Writing – review & editing. DS: Formal analysis, Methodology, Supervision, Validation, Visualization, Writing – review & editing. SS: Formal analysis, Supervision, Visualization, Writing – review & editing. PS: Formal analysis, Methodology, Supervision, Validation, Writing – review & editing. AG: Investigation, Methodology, Project administration, Resources, Supervision, Validation, Visualization, Writing – review & editing. ZQ: Formal analysis, Methodology, Project administration, Resources, Supervision, Visualization, Writing – review & editing.

## Footnotes

^1^https://www.isrctn.com/

^2^www.clinicaltrials.gov

^3^https://trialsearch.who.int/
