# Supplementary figures and images for: Effect of IV ferric carboxy maltose for moderate/severe anemia: a systematic review and meta-analysis
Source: Front Med (Lausanne). 2024 Feb 9;11:1340158. doi: 10.3389/fmed.2024.1340158 (PMC10884292; doi:10.3389/fmed.2024.1340158)

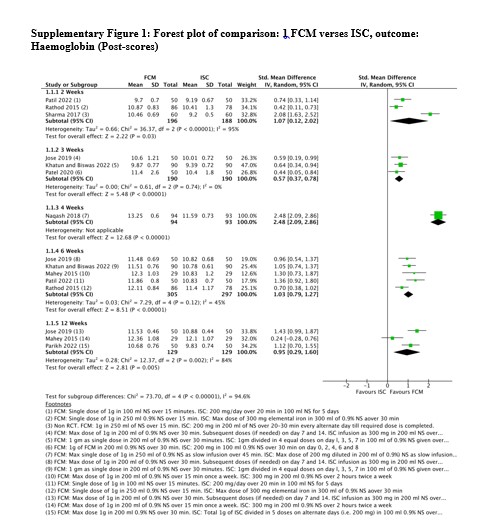

Supplement: Supplementary file 1 [file Image_1.jpg]

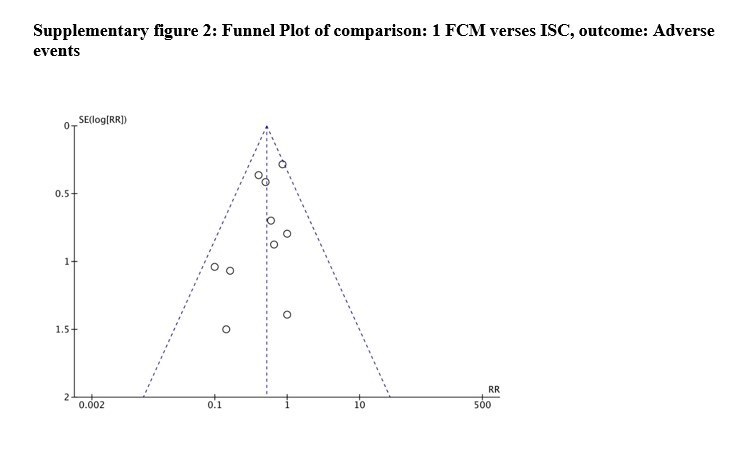

Supplement: Supplementary file 2 [file Image_2.jpg]

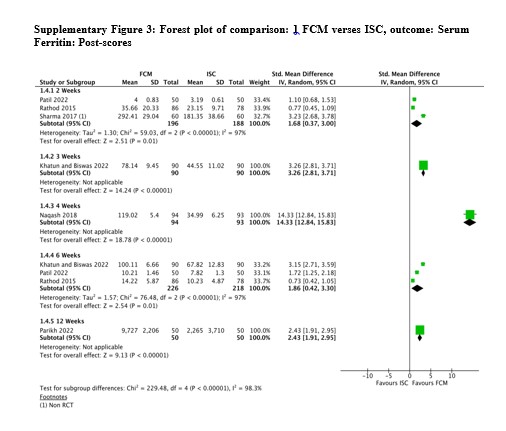

Supplement: Supplementary file 3 [file Image_3.jpg]

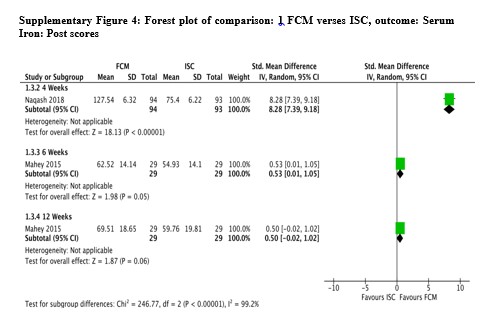

Supplement: Supplementary file 4 [file Image_4.jpg]

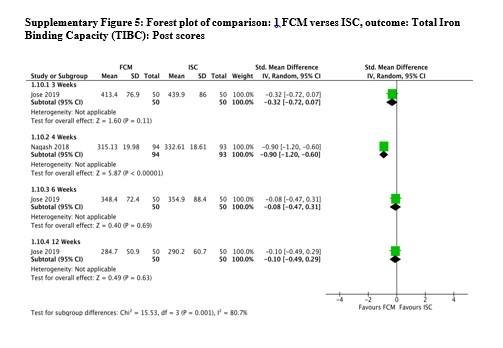

Supplement: Supplementary file 5 [file Image_5.jpg]

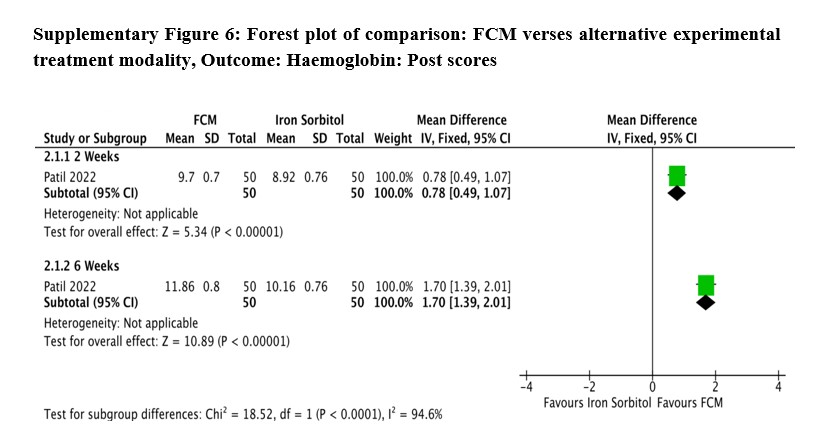

Supplement: Supplementary file 6 [file Image_6.jpg]

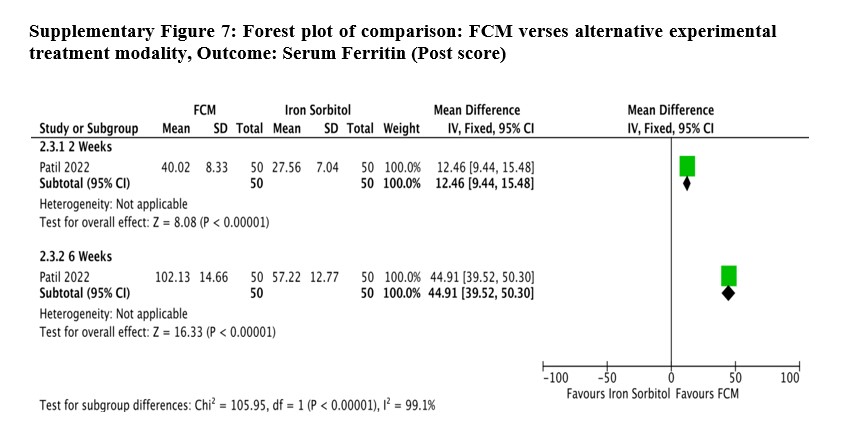

Supplement: Supplementary file 7 [file Image_7.jpg]

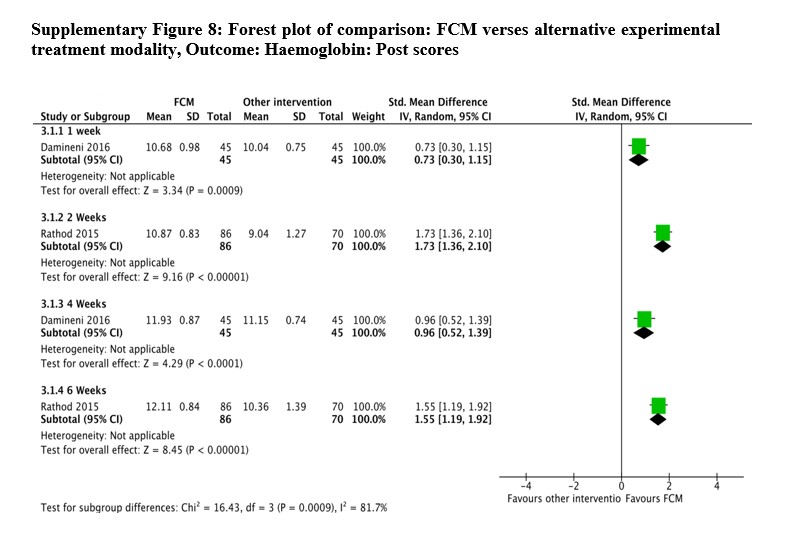

Supplement: Supplementary file 8 [file Image_8.jpg]

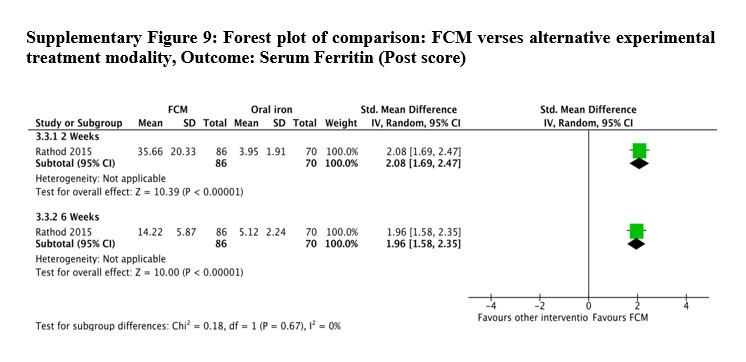

Supplement: Supplementary file 9 [file Image_9.jpg]
