# Supplementary material for: Effect of IV ferric carboxy maltose for moderate/severe anemia: a systematic review and meta-analysis
Source: Front Med (Lausanne). 2024 Feb 9;11:1340158. doi: 10.3389/fmed.2024.1340158 (PMC10884292; doi:10.3389/fmed.2024.1340158)
Supplement: Supplementary file 10 [file Table_1.docx]

Supplementary Table 1: Characteristics of ongoing trials

| **Trial Registration Number** | **Site of Study** | **Title of Study** | **Type of Study** | **Study groups** | **Current status of recruitment** | **Start date and Estimated study completion:** |
| --- | --- | --- | --- | --- | --- | --- |
| ISRCTN14484575 | Not accessible | Comparative study of safety and efficacy of ferric carboxymaltose and iron sucrose in women | Not accessible | Not accessible | Not accessible | Not accessible |
| CTRI/2021/08/036037 | Department of Operational Research, Mumbai, Maharashtra India | A study to assess if using IV Ferric Carboxy Maltose (FCM) in Management of Iron Deficiency Anemia (IDA) among pregnant women is better than IV Iron Sucrose in terms of duration of treatment, side effects and cost-effectiveness at sub district health system in Maharashtra | Parallel Group RCT | FCM verses ISC | Open to Recruitment | Start: 31-08-2021 |
| NCT05358509 | CIFF^[[1]](#footnote-1)^  JNMC^[[2]](#footnote-2)^  SNMC^[[3]](#footnote-3)^  RIMS^[[4]](#footnote-4)^  SMMC^[[5]](#footnote-5)^ | Reducing Anemia in Pregnancy in India: the RAPIDIRON Trial (RAPIDIRON) | 3-arm, open-label, RCT | IV iron formulations (FCM & Iron isomaltoside) verses oral iron | Recruiting | Start: March 15, 2021  Estimated study completion: June 30, 2024 |
| CTRI/2021/08/035672 | Veeda Clinical Research Pvt. Ltd Gujarat | Bioequivalence study of Ferric carboxymaltose solution in patients with iron deficiency anemia, for whom oral iron preparations are ineffective or cannot be used, under fasting condition. | Multicentric, open-label, parallel RCT | FCM of Dr. Reddys Laboratories Ltd, India verses FCM of Vifor Pharma UK in patients with IDA | Open to Recruitment |  |

1. CIFF: Children's Investment Fund Foundation [↑](#footnote-ref-1)
2. JNMC: Jawaharlal Nehru Medical College [↑](#footnote-ref-2)
3. SNMC: S. Nijalingappa Medical College [↑](#footnote-ref-3)
4. RIMS: Raichur Institute of Medical Sciences [↑](#footnote-ref-4)
5. Sawai Mansingh Medical College [↑](#footnote-ref-5)
